# FX5, a non-steroidal glucocorticoid receptor antagonist, ameliorates diabetic cognitive impairment in mice

**DOI:** 10.1038/s41401-022-00884-9

**Published:** 2022-03-08

**Authors:** Dan-yang Zhu, Jian Lu, Rui Xu, Juan-zhen Yang, Xiang-rui Meng, Xing-nan Ou-Yang, Qiu-ying Yan, Rui-fang Nie, Tong Zhao, Yi-di Chen, Yin Lu, Yi-nan Zhang, Wen-jun Li, Xu Shen

**Affiliations:** 1 Jiangsu Key Laboratory for Pharmacology and Safety Evaluation of Chinese Materia Medica and State Key Laboratory Cultivation Base for TCM Quality and Efficacy, Nanjing University of Chinese Medicine, Nanjing, 210023 China; 2 Faculty of Art and Science, Queens University, Kingston, ON K7L 3N6 Canada

**Keywords:** diabetes, diabetic cognitive impairment, glucocorticoid receptor antagonist, FX5, learning and memory, hippocampus, synaptic impairment, neuronal apoptosis, inflammation

## Abstract

Diabetic cognitive impairment (DCI) is a common diabetic complication characterized by learning and memory deficits. In diabetic patients, hyperactivated hypothalamic-pituitary-adrenal (HPA) axis leads to abnormal increase of glucocorticoids (GCs), which causes the damage of hippocampal neurons and cognitive impairment. In this study we investigated the cognition-improving effects of a non-steroidal glucocorticoid receptor (GR) antagonist 5-chloro-N-[4-chloro-3-(trifluoromethyl) phenyl]thiophene-2-sulfonamide (FX5) in diabetic mice. Four weeks after T1DM or T2DM was induced, the mice were administered FX5 (20, 40 mg·kg^−1^·d^−1^, *i.g*.) for 8 weeks. Cognitive impairment was assessed in open field test, novel object recognition test, Y-maze test, and Morris water maze test. We showed that FX5 administration significantly ameliorated the cognitive impairments in both type 1 and 2 diabetic mice. Similar cognitive improvement was observed in diabetic mice following brain GR-specific knockdown by injecting AAV-*si-GR*. Moreover, AAV-*si-GR* injection occluded the cognition-improving effects of FX5, suggesting that FX5 functioning as a non-steroidal GR antagonist. In PA-treated primary neurons (as DCI model in vitro), we demonstrated that FX5 (2, 5, 10 μM) dose-dependently ameliorated synaptic impairment via upregulating GR/BDNF/TrkB/CREB pathway, protected against neuronal apoptosis through repressing GR/PI3K/AKT/GSK3β-mediated tauopathy and subsequent endoplasmic reticulum stress. In LPS-treated primary microglia, FX5 dose-dependently inhibited inflammation through GR/NF-κB/NLRP3/ASC/Caspase-1 pathway. These beneficial effects were also observed in the hippocampus of diabetic mice following FX5 administration. Collectively, we have elucidated the mechanisms underlying the beneficial effects of non-steroidal GR antagonist FX5 on DCI and highlighted the potential of FX5 in the treatment of the disease.

## Introduction

Diabetic cognitive impairment (DCI) is a chronic diabetic complication with complicated pathogenesis [1]. DCI is characterized by deficit of learning and memory abilities. According to the estimates of the International Diabetes Federation, ~463 million people are living with diabetes and the number is expected to rise to 700 million by 2045 [2]. DCI has affected about 30% of diabetic patients, and DCI patients are more likely to suffer from Alzheimer’s disease (AD) compared with common population [3]. Currently, clarifying DCI pathogenesis and performing mechanism-based anti-DCI target and drug research have become hotspots in the field of contemporary diabetes research.

DCI is a multi-factor pathogenic process in patients with both type 1 (T1DM) and type 2 (T2DM) diabetic mellitus, although their mechanisms of cognitive deficits tend to be slightly different. In T1DM pathology, impaired insulin signaling due to insulin deficiency causes inflammation, oxidative stress, apoptosis [4], and neurotrophic factor suppression [5] resulting in synaptic plasticity and integrity impairment and further neuronal loss [6]. By contrast, T2DM has a more complicated pathogenesis, in that impaired insulin signaling induced by insulin resistance impairs tyrosine phosphorylation of IRS leading to inhibition of PI3K/AKT and GSK3β signaling [7] and further activation of tau hyperphosphorylation [8]. Moreover, in addition to impaired insulin signaling, abnormal cholesterol metabolism [9] and excessive activation of hypothalamic-pituitary-adrenal axis (HPA) also aggravates cerebral microvascular lesions [10] and induces neuronal apoptosis, eventually resulting in DCI of T1DM and T2DM.

Glucocorticoids (GCs, e.g., cortisone and hydrocortisone) [11] are a group of hormones secreted by adrenal gland in cellular, molecular, and physiologic network of the body [12]. Under normal physiological conditions, negative feedback is driven by GCs on HPA axis to normalize GCs secretion [13]. However, HPA axis is hyperactivated and circulating cortisol is increased in patients with diabetes [14, 15]. Similarly, adrenal GCs are also elevated in diabetic rodents [16]. GCs render deleterious effects on hippocampus [17], including mitochondrial dysfunction [18], cell cycle arrest and cell death [19]. In addition, abnormal excess of GCs may also induce neuronal atrophy and synaptic dysfunction (e.g., disturbance of integrity of synaptic skeleton) by upregulation of tau hyperphosphorylation [20] and suppression of brain-derived neurotrophic factor (BDNF) [21]. Moreover, chronic GCs exposure increases inflammation in the brain [22]. All results have thus addressed the potency of GCs in the regulation of cognitive impairment.

GCs function by binding glucocorticoid receptor (GR) or mineralocorticoid receptor (MR), two members of nuclear hormone receptors superfamily [23]. MR expression is restricted to a few tissues and GR is ubiquitously expressed in the brain [24]. Currently, several GR antagonists have been reported with beneficial effects on DCI. For example, mifepristone as a steroidal GR antagonist ameliorated cognitive impairment in STZ-induced T1DM rats [25], but its long-term administration may cause side effects like hypokalemia [26], adrenal insufficiency [27], and uterine cramping [28]. Therefore, non-steroidal GR antagonist discovery attracts much attention and reports have been published on the amelioration of non-steroidal GR antagonists on DCI, although the detailed mechanisms are still obscure [29].

**Fig. 1:**
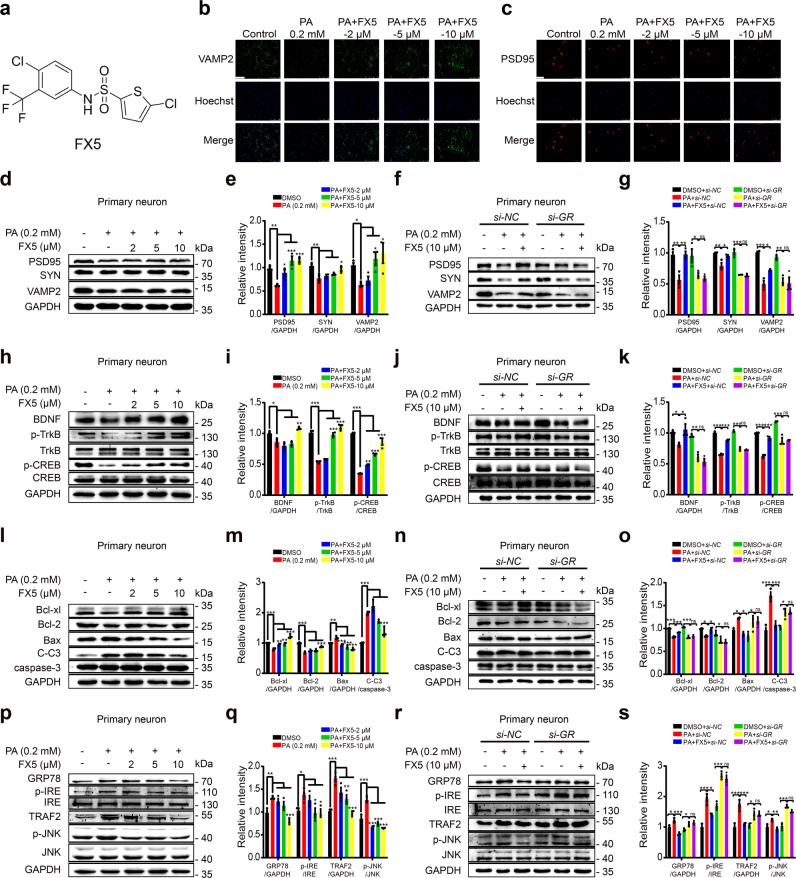
FX5 ameliorated synaptic impairment and protected against neuronal apoptosis and ER stress in PA-treated primary neurons. **(a)** Chemical structure of FX5. Immunostaining assays for synapse-related proteins (**b**) VAMP2 (Scale bar: 250 μm) and (**c**) PSD95 (Scale bar: 50 μm) in PA-treated primary neurons (*n* = 4). Immunoblot analysis with quantifications showed that (**d**, **e**) FX5 antagonized the PA-induced suppression of synapse-related proteins (PSD95, SYN, and VAMP2) and (**f**, **g**) *si-GR* deprived FX5 of its antagonistic capability in PA-treated primary neurons (*n* = 3). Immunoblot analysis with quantifications showed that (**h**, **i**) FX5 antagonized PA-induced repression on the protein levels of BDNF, p-TrkB and p-CREB and (**j**, **k**) *si-GR* deprived FX5 of its antagonistic capability in PA-treated primary neurons (*n* = 3). Immunoblot analysis with quantifications showed that (**l**, **m**) FX5 antagonized PA-induced decrease in anti-apoptotic proteins (Bcl-2 and Bcl-xl) and increase in pro-apoptotic proteins (Bax and C-C3), and (**n**, **o**) *si-GR* deprived FX5 of its antagonistic capability in PA-treated primary neurons (*n* = 3). Immunoblot analysis with quantifications showed that (**p**, **q**) FX5 inhibited PA-induced ER stress and (**r**, **s**) *si-GR* deprived FX5 of its inhibitory capability in PA-treated primary neurons (*n* = 3). GAPDH was used as loading control. *n* = 3 independent experiments. Mean ± SEM. One-way ANOVA followed by Dunnett’s multiple comparison test. *P < 0.05, **P < 0.01, ***P < 0.001 vs PA group.

In the current work, we determined that small molecule 5-chloro-N-[4-chloro-3- (trifluoromethyl)phenyl] thiophene-2-sulfonamide (FX5, Fig. 1a [30]) as a non-steroidal GR antagonist improved learning and memory in both T1DM and T2DM mice. The underlying mechanisms have been intensively investigated by assay against the GR-specific knockdown diabetic mice treated with adeno-associated virus (AAV)-ePHP-*si-GR*. Our work has strongly addressed the potency of non-steroidal GR antagonist in the amelioration of DCI and highlighted the potential of FX5 in the treatment of this disease.

## Materials and methods

### Materials

All cell culture reagents were purchased from Gibco (Grand Island, New York, USA). Plasmids of pCI-nGFP-C656G, pRL-SV40 and pUAS-TK-Luc were kindly donated respectively by Dr. Gordon Hager (National Cancer Institute, National Institutes of Health), Dr. J Larry Jameson (Department of Medicine, Northwestern Memorial Hospital) and Dr. Daniel P. Kelly (School of Medicine, Washington University). Other plasmids were available in our own lab. Dimethy1 sulfoxide (DMSO), Tween-80, 3-(4,5-dimethylthiazol-2-yl)-2,5-diphenyltetrazolium bromide (MTT), DNase, poly-*D*-lysine (PDL), dexamethasone (Dex), corticosterone (Cort), progesterone (Prog), T0901317 (T090), estradiol (Est), streptozotocin (STZ), secondary anti-rabbit/mouse antibodies, lipopolysaccharide (LPS), and palmitic acid (PA) were purchased from Sigma-Aldrich (St, Louis, MO, USA). RIPA buffer and BCA protein assay kits were purchased from Beyotime (Shanghai, China). Protease inhibitor cocktail and phosphatase inhibitor cocktail were purchased from Thermo Fisher Scientific (Waltham, MA, USA). si-negative control (*si-NC*) and *si-GR* plasmids were purchased from GenePharma (Shanghai, China). Lipofectamine 2000 was purchased from Invitrogen (Carlsbad, CA, USA).

### Cell culture

Pregnant mice and newborn mice were purchased from Vital River Laboratory Animal Technology Co (Beijing, China). All animal experiments were performed following the institutional ethical guidelines on animal care of Nanjing University of Chinese Medicine.

Primary neurons were prepared from embryonic day 16 to 18-mouse brains. The brain tissues were minced into small pieces, digested with D-Hank’s buffer containing 0.125% trypsin and 200 U/mL DNase, and incubated for 15 min at 37 °C. Digestion was stopped by adding 4 mL DMEM supplemented with 10% FBS. The cell fluid was then diluted at a density of 6 × 10^5^ cells/mL on PDL-coated cell culture plates. After 6 h, the medium was replaced by neurobasal medium supplemented with 2% B27, 0.5 μM-glutamine and 50 U/mL penicillin-streptomycin [31].

Primary microglia were prepared from newborn mice within 24 h. The brain tissues were minced into small pieces, and digested with Hank’s buffer containing 0.25% trypsin and 200 U/mL DNase. The cell suspension was incubated for 10 min at room temperature, while digestion was stopped by adding 4 mL DMEM supplemented with 10% FBS. Then, the dissociated cells were cultured in DMEM/F12 supplemented with 10% FBS and 50 U/mL penicillin-streptomycin using a PDL-coated 75 cm^2^ flask at a density of 2 × 10^5^ cells/cm^2^. After 7 days, the microglia were dissociated by shaking the flasks several times and harvested by centrifugation at 1500 r/min for 10 min, followed by dilution at a density of 5 × 10^4^ cells/mL on PDL-coated cell culture plates [31].

Human embryonic kidney HEK293T cells and U2OS/GR-GFP cells were cultured in DMEM supplemented with 10% FBS and 100 U/mL penicillin-streptomycin.

All cells were cultured in a humidified incubator with 5% CO_2_ at 37 °C.

### Mammalian transactivation assay

Mammalian transactivation assay was carried out to detect the antagonistic effect of FX5 on GR transcriptional activity according to the reported approach [32]. Briefly, HEK293T cells were seeded in 48-well plates at a density of 5 × 10^4^ cells/well and cultured overnight. Cells were then co-transfected with plasmids of pCI-nGFP-C656G, pGL3-GRE-Luc, and pRL-SV40 by calcium phosphate cell transfection kit (Beyotime, Shanghai, China). After 6 h, cells were incubated with Dex (10 nM) or/and FX5 (10 μM) for 24 h. Finally, firefly and Renilla luciferase activities were measured by dual-luciferase reporter assay system kit (Promega, Madison, WI, USA).

The effect of FX5 on other nuclear receptors MR, progesterone receptor (PR), liver X receptor β (LXRβ), and estrogen receptor β (ERβ) were also detected by using mammalian transactivation assay.

### Nuclear translocation assay

U2OS/GR-GFP cells were seeded in 96-well plates at a density of 1 × 10^4^ cells/well and cultured overnight. The cells were then incubated with PA (0.2 mM) or/and different concentrations of FX5 (2, 5, or 10 μM) for 24 h. After incubation, cells were immobilized with 4% paraformaldehyde, and nucleus was dyed with Hoechst 33342 (Solarbio, Beijing, China). Fluorescence pictures were obtained using Leica fluorescence microscope (Leica Microsystems, Nussloch, Germany). The ratio of GR nuclear translocation was calculated by ImageJ software.

### MTT assay

Viability of the cultured cells was determined by MTT assay [33]. Briefly, primary neurons were seeded in 96-well plates at a density of 1 × 10^5^ cells/well and cultured overnight. The cells were then incubated with different concentrations of FX5 (2, 5, or 10 μM) for 24 h, and 0.5 mg/mL MTT was added into the medium for additional 4 h. After incubation, 100 μL DMSO was added to dissolve formazan crystals, and absorbance at 490 nm was measured using a Spectra Max i3x reader (Molecular Devices, Sunnyvale, CA, USA).

### Animals

All animal experiments were performed following the institutional ethical guidelines on animal care of Nanjing University of Chinese Medicine. All mice were maintained under standard conditions at room temperature (22 °C) by a 12 h light/dark cycle. Four (for establishing T2DM model) and 8 (for establishing T1DM model)-week-old C57BL/6 male mice were purchased from Vital River Laboratory Animal Technology Co (Beijing, China).

#### T1DM mice

8-week-old C57BL/6 male mice were fed for 7 days to acclimatize and then received a single *i.p*. injection of 150 mg/kg STZ in 0.5 M sodium citrate buffer or vehicle. One week after injection, mice with blood glucose level higher than 16.7 mM were classified as T1DM (STZ) mice [34].

#### T2DM mice

4-week-old C57BL/6 male mice were fed for 7 days to acclimatize and then divided into normal diet group and high-fat diet (HFD) group. The normal diet group mice were fed with a standard laboratory diet and the HFD group mice were fed with a formulation comprising 60 kcal% fat for 4 weeks to induce insulin resistance. All mice were then fasted for 6 h and the HFD group mice received an *i.p*. injection of 100 mg/kg STZ, and normal group mice received the same volume of vehicle. T2DM (HFD/STZ) mice were identified as those with blood glucose level higher than 11.1 mM after 1 week [35].

#### AAV-induced GR knockdown mice

AAV-ePHP-*si-GR* (AAV-*si-GR*) [31] and negative control vector (AAV-*si-NC*) were purchased from Shanghai Genechem Co (Shanghai, China). The titer of AAV-*si-GR* was 3.14 × 10^13^ μg/mL. AAV-induced GR knockdown diabetic mice (STZ + AAV-*si-GR*; HFD/STZ + AAV-*si-GR*) were obtained via tail vein injection with a dose of 1 × 10^11^ μg/mouse [36] 2 weeks after diabetic model was established.

The knockdown efficiency of AAV injection was detected 2 weeks after diabetic model was established (Supplementary Fig. S1a–d).

### Animal administration

Diabetic mice were randomized into diabetic group and FX5 (20, 40 mg/kg) group. Four weeks after diabetic model was established, the animals occurred cognitive impairment as reported [37]. Diabetic mice in FX5 groups were administered FX5 by gastric perfusion, and mice in control (C57BL/6 male mice) and model group were given the same amount of vehicle (2% DMSO, 5% Tween-80).

AAV-injected diabetic mice were randomized into AAV-*si-NC* group, AAV-*si-GR* group, and AAV-*si-GR* + FX5 (40 mg/kg) group. After 2 weeks, FX5 and vehicle were administered by gastric perfusion.

Mice administrations were performed for 8 weeks, once a day. *n* = 25 for T1DM/group, and *n* = 15 for T2DM/group.

### Open field test

The open field test (OFT) is a straightforward test to evaluate the locomotion and exploratory behavior in mice [38]. Before OFT testing, mice were transferred to the testing room and acclimated for at least 1 h. Mice were then tested in a clear plastic chamber (32 cm × 32 cm × 20 cm) for 15 min. The apparatus was cleaned with 70% alcohol after testing of each mouse. Total movement in the open field and the time spent in center were recorded for data analysis.

### Novel object recognition test

Novel object recognition (NOR) test is based on rodent’s natural curiosity to explore the immediate environment [39]. The test consists of four sequential daily trials. During the habituation trial, mice were placed in the center of the open-field apparatus and allowed to explore freely for 15 min. During the acquisition trial, each animal was given 5 min to freely explore two identical objects (Object A) and then returned to the home cage. After 4 h, mice were returned to the test arena where one of the sample objects had been replaced by a novel one (Object B). The apparatus and objects were cleaned with 70% ethanol before use and between each animal test. All data were collected for animal performance analysis. During the test phase, a discrimination index was calculated.

### Y-maze test

As described previously [40], the Y-maze (46 cm × 11 cm × 25 cm) consists of three arms (A, B and C) at 120° angles to each other. Before testing, mice were allowed to habit in the testing room for 30 min. For the training trials, one arm of the maze was blocked off (novel arm, N). The mice were placed in the intersection of three arms and allowed to move freely in the other two arms (other arm, O) for 5 min. Two hours later, the blocked arm was opened, and the mice were allowed to explore again in the three arms for 5 min. A mouse was considered to have entered an arm when all four paws were positioned in the arm runway. Total arm entries were measured and the percentage number of entries into N was calculated as follows: %N = (100% × N)/(N + O).

### Morris water maze test

Morris water maze (MWM) test was performed according to the published approach [41]. Briefly, for training stage, mice were trained to find an invisible submerged platform placed in the circular pool (120 cm in diameter, 50 cm deep) filled with white food additives. Mice were given 60 s to find the platform and allowed to stay at the platform for 10 s. If a mouse failed to find the platform within 60 s, it was pulled gently to the platform and kept there for 10 s. For testing stage, the platform was removed. Mice were given 60 s to search for the platform. All data were collected for animal performance analysis. In data analysis, the pool was artificially divided into four equal quadrants formed by imaging lines, intersected in the center of the pool at right angles by north, south, east, and west.

### Golgi staining

Morphology of dendritic spines in the brain was analyzed using a FD Rapid Golgi Stain Kit (FD Neuro technologies, Elliot City, MD, USA) by manufacturer’s instructions. Images for neurons were obtained with an automated upright microscope (Leica Microsystems, Nussloch, Germany).

### Electrophysiology test

Long-term potentiation (LTP) assay was performed by published approach [31]. In the assay, the baseline was recorded for 10 min, and the LTP in the DG area was then induced by four trains of 100 Hz stimuli with the same intensity of the test stimulus. The strength of synaptic transmission was determined by measuring the initial (20%–80% rising phase) slope of field excitatory postsynaptic potentials (fEPSPs).

### Immunoblot assay

The assay was conducted based on the published method [42].

#### Cell-based assay

Primary neurons were incubated with PA (0.2 mM) or/and different concentrations of FX5 (2, 5, or 10 μM) for 24 h and primary microglia were incubated with LPS (100 ng/mL) or/and different concentrations of FX5 (2, 5, or 10 μM) for 24 h, and then lysed with RIPA buffer containing protease inhibitor cocktail and phosphatase inhibitor cocktail on ice for 20 min, followed by centrifugation at 12,000 r/min for 30 min at 4 °C.

#### Brain tissue-based assay

Brain tissues of mice in each group were homogenized with RIPA buffer containing protease inhibitor cocktail and phosphatase inhibitor cocktail by a hand-hold motor and kept on ice for 1 h. After the tissues were lysed completely, the homogenates were centrifuged at 12,000 r/min for 30 min at 4 °C.

The supernatants were collected, and protein concentrations were determined using BCA protein assay kit. Proteins were mixed with 2× SDS-PAGE sample buffer and then boiled for 15 min at 95 °C.

Cell or tissue extracts were separated by SDS-PAGE and transferred to a nitrocellulose filter membrane (GE Healthcare, Waukesha, WI, USA). After blocking for 2 h at room temperature, the membranes were incubated with corresponding antibodies (dilution 1:1000) overnight at 4 °C. The membranes were washed and incubated with secondary anti-rabbit/mouse antibodies (dilution 1:3000) conjugated with horseradish peroxidase for 2 h at room temperature. The blots were developed and visualized using a ChemiDoc MP (Bio-Rad, Hercules, CA, USA).

The details of antibodies were provided in Supplementary Table S1.

### RNA isolation and quantitative real-time PCR

Total RNA in cells and brain tissues was extracted by RNAiso plus regent according to the manufacturer’s protocol [43], and 1 μg total RNA was reverse-transcribed into cDNA using Prime Script RT reagent kit (Takara Biomedical Technology Co, Beijing, China). Finally, mRNA levels of different genes were quantified by quantitative real-time PCR using SYRB Premix Ex Taq kit and normalized to GAPDH or β-Actin. The thermal cycling condition was 95 °C for 10 s followed by 40 cycles of amplification at 95 °C for 10 s, 60 °C for 20 s, 72 °C for 10 s and 80 °C for 1 s, then keeping at 72 °C for 10 min.

The primers used in real-time PCR were shown in Supplementary Table S2.

### *siRNA* plasmid transfection

Primary neurons or primary microglia were transfected with 50 nM *si-NC* or *si-GR*. *si-NC* or *si-GR* was dissolved in 25 μL of Opti-MEM and mixed with Lipofectamine 2000 transfection reagent for 15 min, then cells were incubated with the mixture. After 6 h, the medium was changed into DMEM or DMEM/F12 and incubated for 24 h. *si-NC* was used as a negative control.

### Immunostaining

#### Synaptic integrity analysis

Primary neurons were incubated with PA (0.2 mM) or/and different concentrations of FX5 (2, 5, or 10 μM) for 24 h, then primary neurons grown on slides were washed three times with PBS and fixed in 4% paraformaldehyde for 5 min at room temperature. The cells were then incubated with 0.3% Triton X-100 for 10 min and blocked with 4% BSA for 30 min at room temperature, followed by incubation with VAMP2 or PSD95 antibodies (dilution 1:500) overnight at 4 °C. The slide was washed three times and incubated with fluorescent secondary detection antibodies (dilution 1:250) goat anti-rabbit and goat anti-mouse for 1 h and the nucleus was stained with Hoechst 33342.

#### Brain tissues

The left hemisphere was fixed in 4% paraformaldehyde overnight and kept in 30% sucrose until sank to the bottom. The tissues were embedded in OCT and sectioned by using a cryostat microtome. For detecting the expression of GR, MAP2, Iba1, NF-κB and NLRP3, the slides were incubated in 5% Triton X-100 for 10 min and blocked in 5% BSA for 30 min at room temperature, followed by incubation overnight at 4 °C with primary antibody (dilution 1:400). The slides were then washed three times and incubated with fluorescent secondary detection antibodies (dilution 1:250) for 1 h and the nucleus was stained with Hoechst 33342.

The antibodies used for immunostaining were provided in Supplementary Table S3. Images were acquired using a Leica fluorescence microscope and analyzed by ImageJ software.

### DAB staining

Brain slides (20 μm) were incubated in 5% Triton X-100 for 10 min and blocked in 5% BSA for 30 min at room temperature, followed by incubation with AT8 antibody (Invitrogen, St, Louis, MO, USA) (dilution 1:250) overnight at 4 °C. Then the slides were washed three times and incubated with anti-mice secondary antibody for 1 h and subsequently with diaminobenzidine for 5 min and washed three times [44]. Images were obtained with an automated upright microscope.

### Nissl staining

The slides were washed twice and incubated in Cresyl violet Stain for 30 min at 37 °C. Slides were washed with ddH_2_O for 5 s and placed in Nissl differentiation for several seconds to 2 min until the background of the slides was close to achromatic. Then the slides were dehydrated in ethanol for 5 s. Finally, xylene solution was dropped to the slides sealed with neutral gum [45]. Images were obtained with an automated upright microscope.

### Statistical analysis

Data were presented as mean ± SEM. Unpaired two-tailed Student’s *t* test was used for two-group comparison. One-way ANOVA with Dunnett’s post-test was used for at least three group’s comparisons. Significance was defined as *P* < 0.05.

## Results

### FX5 ameliorated synaptic impairment through GR/BDNF/TrkB/CREB pathway in PA-treated primary neurons

FX5 was determined as a non-steroidal GR antagonist by mammalian one-hybrid and transactivation assays as well as nuclear translocation assay in our previous work [30]. Notably, the antagonistic activity of FX5 against other nuclear receptors was also evaluated by mammalian transactivation assay. The results indicated that the antagonism of FX5 against GR (60%) was obviously superior to that of MR (27%), PR (27%), LXRβ (28%) and ERβ (29%) (Supplementary Fig. S1e–i).

Here, we investigated the potential of FX5 in ameliorating DCI. Since MTT assay result demonstrated that FX5 rendered no influence on primary neuron viability at 2, 5 and 10 μM (Supplementary Fig. S1j), these three concentrations were set in the following experiments.

PA is a common fatty acid in human diet and the plasma PA level is elevated in diabetic patients [46]. PA is widely used to establish AD-like model by inducing tau hyperphosphorylation [47], apoptosis [48], and ER stress [46]. Moreover, increased tissue PA concentrations may also increase GCs concentrations [49]. In our study, immunostaining results demonstrated that PA increased GR expression and promoted GR nuclear translocation in U2OS/GR-GFP cells, which strengthened the feasibility of PA for establishing DCI model in vitro. In addition, FX5 antagonized PA-induced GR expression and GR nuclear translocation (Supplementary Fig. S1k–m).

#### FX5 protected synaptic integrity by antagonizing GR

Given that synaptic loss is responsible for DCI [50], immunostaining and immunoblot assays were performed to inspect the potential of FX5 in protecting synaptic integrity by detecting synaptic integrity-related proteins PSD95, synaptophysin (SYN) and VAMP2 in primary neurons. As shown in Fig. 1b–e, FX5 antagonized PA-induced suppression of these proteins. Moreover, *si*-*GR* deprived FX5 of its above capability (Fig. 1f, g). All results demonstrated that FX5 protected synaptic integrity by antagonizing GR in PA-treated primary neurons.

#### FX5 upregulated BDNF/TrkB/CREB pathway by antagonizing GR

Given that BDNF/TrkB/CREB signaling functions potently in the regulation of synapse plasticity [51], we inspected the potential regulation of FX5 against this pathway in PA-treated primary neurons. Immunoblot results demonstrated that FX5 antagonized PA-induced repression of the protein levels of BDNF, p-TrkB and p-CREB (Fig. 1h, i), and *si*-*GR* deprived FX5 of its antagonistic ability (Fig. 1j, k). These results indicated that FX5 upregulated BDNF/TrkB/CREB pathway by antagonizing GR.

Thus, all results demonstrated that FX5 ameliorated synaptic impairment through GR/BDNF/TrkB/CREB pathway in PA-treated primary neurons.

### FX5 protected against neuronal apoptosis by repressing GR/PI3K/AKT/GSK3β pathway-mediated tauopathy and subsequent endoplasmic reticulum (ER) stress in PA-treated primary neurons

#### FX5 protected against neuronal apoptosis by antagonizing GR

Brain nerve injury and neuronal apoptosis are important pathological changes of DCI [52]. We performed immunoblot assay and the results indicated that FX5 antagonized the PA-induced downregulation of anti-apoptotic proteins (Bcl-xl and Bcl-2) and upregulation of pro-apoptotic proteins (cleaved caspase-3 (C-C3) and Bax) (Fig. 1l, m), while *si*-*GR* deprived FX5 of its anti-apoptotic capability (Fig. 1n, o) in PA-treated primary neurons. All results implied that FX5 protected against neuronal apoptosis by antagonizing GR.

#### FX5 repressed ER stress by antagonizing GR

ER stress was tightly implicated in cognitive impairment [53] and accumulated unfolded proteins in ER promoted dissociation of ER chaperone GRP78 from IRE, causing IRE interaction with TRAF2 and further activation of downstream target JNK ultimately resulting in apoptosis [54]. Immunoblot assay was next performed in PA-treated primary neurons. The results demonstrated that FX5 inhibited PA-induced ER stress by antagonizing the upregulation of protein levels of GRP78, p-IRE, TRAF2, and p-JNK (Fig. 1p, q). Moreover, *si*-*GR* deprived FX5 of its above-mentioned antagonistic capabilities (Fig. 1r, s), implying that FX5 repressed ER stress by targeting GR.

#### FX5 attenuated tau hyperphosphorylation by antagonizing GR

**Fig. 2:**
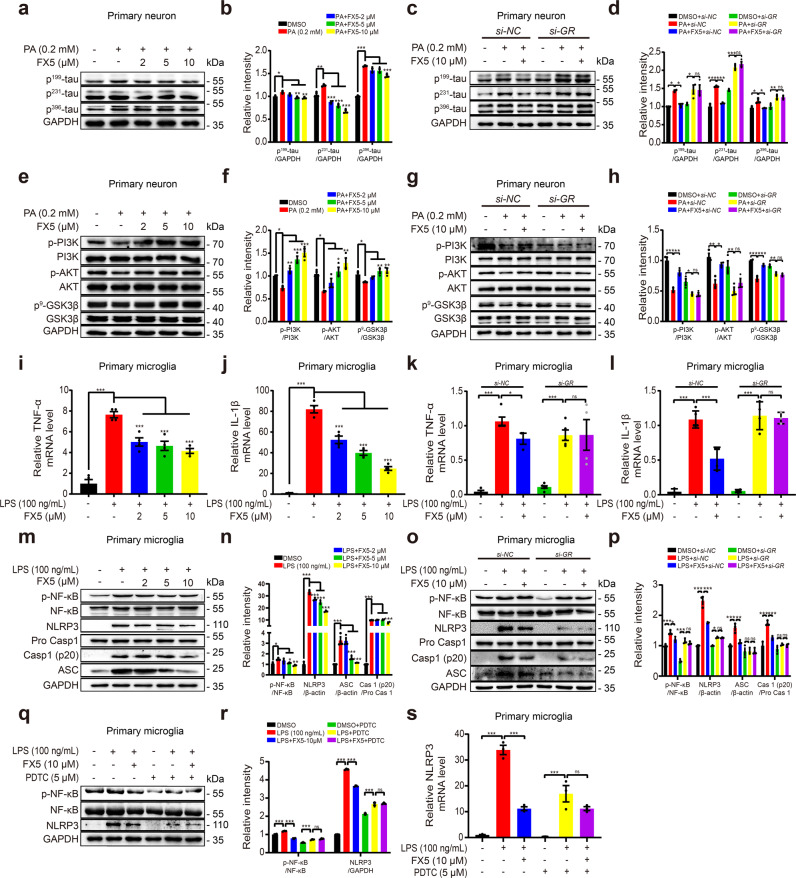
FX5 attenuated tauopathy in PA-treated primary neurons and repressed inflammation in LPS-treated primary microglia. Immunoblot analysis with quantifications showed that (**a**, **b**) FX5 antagonized PA-stimulated tau hyperphosphorylation at Ser199, Thr231 and Ser396 and (**c**, **d**) *si-GR* deprived FX5 of its antagonistic capability in PA-treated primary neurons (*n* = 3). Immunoblot analysis with quantifications showed that (**e**, **f**) FX5 antagonized PA-induced decline in phosphorylation levels of PI3K, AKT (Ser 473) and GSK3β (Ser 9) and (**g**, **h**) *si-GR* deprived FX5 of its antagonistic capability in PA-treated primary neurons (*n* = 3). RT-PCR results demonstrated that (**i**, **j**) FX5 antagonized LPS-stimulated expression levels of pro-inflammatory factors TNF-α and IL-1β, and (**k**, **l**) *si-GR* deprived FX5 of its antagonistic capability in LPS-treated primary microglia (*n* = 4). Immunoblot analysis with quantifications showed that (**m**, **n**) FX5 antagonized LPS-induced upregulation of p-NF-κB, NLRP3, ASC and Caspase-1 (P20), and (**o**, **p**) *si-GR* deprived FX5 of its antagonistic capability in LPS-treated primary microglia (*n* = 3). (**q**) Immunoblot analysis with (**r**) quantifications and (**s**) RT-PCR result showed that treatment of NF-κB inhibitor PDTC abolished FX5-induced suppression against NLRP3 in LPS-treated primary microglia (*n* = 3). GAPDH was used as loading control. *n*=3 independent experiments. Mean ± SEM. One-way ANOVA followed by Dunnett’s multiple comparison test. *P < 0.05, **P < 0.01, ***P < 0.001 vs PA or LPS group.

Considering that tau hyperphosphorylation is highly contributable to neurofibrillary tangles formation in brain with DCI [55], and accumulation of the hyperphosphorylated tau as a misfolded protein in the ER could cause neurodegeneration due to protracted ER stress [56, 57], immunoblot assay was detected and the results demonstrated that FX5 antagonized PA-stimulated tau hyperphosphorylation at Ser199, Thr231 and Ser396 in primary neurons (Fig. 2a, b). Notably, *si*-*GR* deprived FX5 of its antagonistic capability against tau hyperphosphorylation (Fig. 2c, d). These results thus demonstrated that FX5 attenuated tau hyperphosphorylation by antagonizing GR.

#### FX5 regulated PI3K/AKT/GSK3β pathway by antagonizing GR

Since activated PI3K induces AKT activation to phosphorylate varied substrates including GSK3β, and PI3K/AKT signaling dysfunction activates GSK3β leading to tau hyperphosphorylation [8], we examined the regulation of FX5 against the protein levels of p-PI3K, p-AKT (Ser 473) and p-GSK3β (Ser 9) in PA-treated primary neurons. Immunoblot results indicated that FX5 antagonized PA-induced decline in these proteins (Fig. 2e, f), and *si*-*GR* deprived FX5 of its antagonistic capability against PI3K/AKT/GSK3β pathway (Fig. 2g, h). Thus, these results demonstrated that FX5 regulated PI3K/AKT/GSK3β pathway by antagonizing GR.

Together, all results demonstrated that FX5 protected against neuronal apoptosis by repressing GR/PI3K/AKT/GSK3β pathway-mediated tauopathy and subsequent ER stress in PA-treated primary neurons.

### FX5 repressed inflammation through GR/NF-κB/NLRP3/ASC/Caspase-1 pathway in LPS-treated primary microglia

#### FX5 repressed inflammation by antagonizing GR

As inflammation contributes highly to DCI [58], we detected the potential inhibitory capability of FX5 against inflammation in LPS-treated primary microglia by RT-PCR. The results indicated that FX5 antagonized LPS-induced upregulation of pro-inflammatory factors TNF-α and IL-1β (Fig. 2i, j), and *si*-*GR* deprived FX5 of its antagonistic capability to these pro-inflammatory factors (Fig. 2k, l). These results thus implied that FX5 repressed inflammation by antagonizing GR.

#### FX5 repressed NF-κB/NLRP3/ASC/Caspase-1 pathway by antagonizing GR

NF-κB and NLRP3 are tightly implicated in inflammation [59], and NLRP3 inflammasome becomes activated when exposed to stimuli, in that NLRP3 undergoes a conformational change and assembles with adaptor protein ASC and pro-Caspase 1 resulting in activation of Caspase 1 and further formation of mature IL-1β [60]. With these facts, immunoblot assay was performed in primary microglia and the results indicated that FX5 antagonized LPS-induced upregulation of p-NF-κB, NLRP3, ASC and Caspase-1 (P20) (Fig. 2m, n), and *si*-*GR* deprived FX5 of its antagonistic capability against NLRP3 inflammasome (Fig. 2o, p).

Given the potent regulation of NF-κB against NLRP3 inflammasome [59], we further investigated whether NF-κB signaling was required for NLRP3 regulation by using NF-κB inhibitor PDTC in the assay [61]. As shown in Fig. 2q–s, PDTC blocked the capability of FX5 in antagonizing LPS-induced upregulation of protein and mRNA levels of NLRP3 in primary microglia. These results implied that FX5 repressed NF-κB/NLRP3/ASC/Caspase-1 pathway by antagonizing GR.

Therefore, all results demonstrated that FX5 repressed inflammation through GR/NF-κB/NLRP3/ASC/Caspase-1 pathway in LPS-treated primary microglia.

### FX5 ameliorated DCI in mice by antagonizing GR

**Fig. 3:**
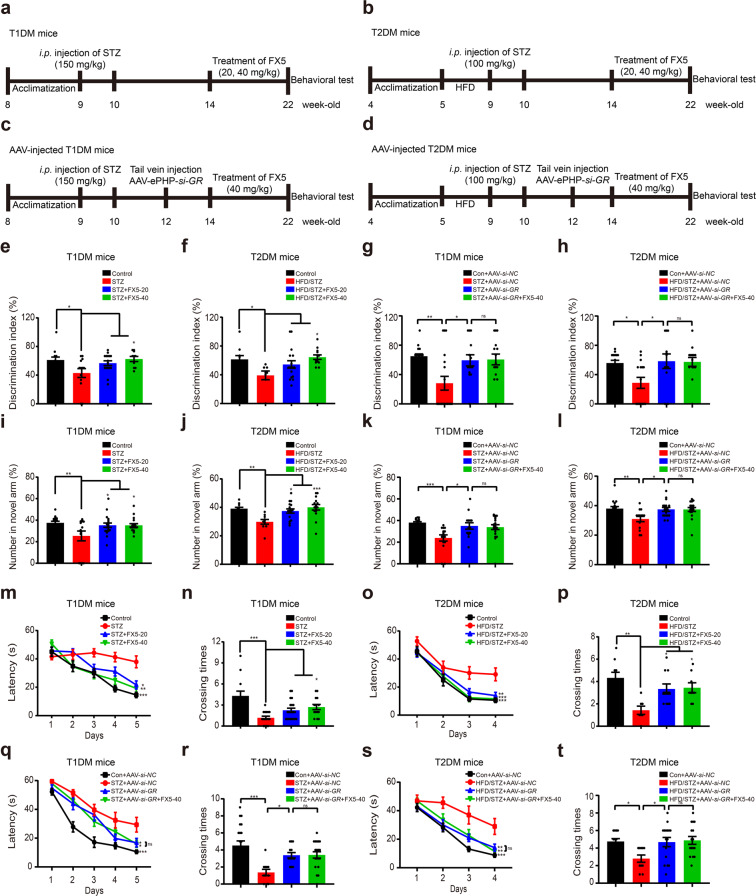
FX5 ameliorated DCI in mice by antagonizing GR. Schedules of animal treatment and behavior test to (**a**, **c**) STZ and (**b**, **d**) HFD/STZ mice. NOR test results indicated that FX5 (20, 40 mg/kg) or AAV-*si-GR* treatment ameliorated short-term working memory defects in (**e**, **g**) STZ and (**f**, **h**) HFD/STZ mice (*n* ≥ 8 per group), and FX5 (40 mg/kg) treatment had no impacts on such defects in (**g**) STZ + AAV-*si-GR* and (**h**) HFD/STZ + AAV-*si-GR* mice (*n* ≥ 12 per group). Y-maze test results indicated that FX5 (20, 40 mg/kg) or AAV-*si-GR* treatment ameliorated spatial working memory defects in (**i**, **k**) STZ and (**j**, **l**) HFD/STZ mice (*n* ≥ 10 per group), and FX5 (40 mg/kg) treatment had no impacts on such defects in (**k**) STZ + AAV-*si-GR* and (**l**) HFD/STZ + AAV-*si-GR* mice (*n* ≥ 14 per group). Escape latency results during platform trials indicated that FX5 (20, 40 mg/kg) or AAV-*si-GR* treatment ameliorated learning and memory dysfunction in (**m**, **q**) STZ and (**o**, **s**) HFD/STZ mice (*n* ≥ 7 per group), and FX5 (40 mg/kg) treatment had no impacts on such dysfunctions in (**q**) STZ + AAV-*si-GR* and (**s**) HFD/STZ + AAV-*si-GR* mice (*n* ≥ 12 per group). Times of platform crossing in probe trials for (**n**) STZ, (**p**) HFD/STZ, (**r**) STZ + AAV-*si-NC* and (**t**) HFD/STZ + AAV-*si-NC* mice (*n* ≥ 7 per group). Mean ± SEM. One-way ANOVA followed by Dunnett’s multiple comparison test. *P < 0.05, **P < 0.01, ***P < 0.001 *vs* model or AAV-*si-NC* treated model group.

To further investigate the capability of FX5 in ameliorating DCI, the cognitive ability of diabetic mice was evaluated by OFT, NOR, Y-maze, and MWM tests with FX5 (20, 40 mg/kg) treatment. Moreover, to confirm that FX5 ameliorated DCI in mice by antagonizing GR, the related assays were performed in AAV-*si-GR-*injected diabetic mice with brain GR-specific knockdown (Fig. 3a, d).

#### OFT test

OFT test was used to investigate the autonomous and exploratory behaviors of mice in a new environment [62]. As indicated in Supplementary Fig. S2a–d, FX5-treated (STZ + FX5; HFD/STZ + FX5) or AAV-*si-GR*-injected (STZ + AAV-*si-GR*; HFD/STZ + AAV-*si-GR*) diabetic mice spent more time in the center compared with vehicle-treated (STZ; HFD/STZ) diabetic mice. In addition, no significant difference was found in the totally moved distance among the mice in all groups (Supplementary Fig. S2e–h).

#### NOR test

This test was to evaluate the short-term working memory of mice [63]. As indicated in Fig. 3e–h, FX5 or AAV-*si-GR* treatment increased the discrimination index of diabetic mice.

#### Y-maze test

This test was to examine spatial working memory of mice [64]. As expected, the percentage of number in novel arms of FX5 or AAV-*si-GR*-treated diabetic mice was increased compared with that in vehicle-treated diabetic mice (Fig. 3i–l).

In addition, the result that there was no difference in total arm entries of T2DM mice in all groups (Supplementary Fig. S2j and l) indicated no deficit in motor function of T2DM mice, while FX5 or AAV-*si-GR* treatment failed to ameliorate the total arm entries in T1DM mice (Supplementary Fig. S2i and k). These results implied that the motor ability of T1DM mice was more severely impaired than that of T2DM.

#### MWM test

MWM test was typically to examine spatial learning and long-term memory of mice [65]. As indicated in Fig. 3m, o, q and s, FX5 or AAV-*si-GR-*treated diabetic mice spent shorter time in finding the hidden platforms in terms of daily escape latency through 5-day (for STZ mice) or 4-day (for HFD/STZ mice) consecutive training compared with vehicle-treated diabetic mice. The different training days for diabetic mice reflected that the cognitive impairment of T1DM is more serious than that of T2DM. Testing trails were conducted after the training and the results demonstrated that FX5 or AAV-*si-GR*-treated diabetic mice crossed the target quadrant more frequently than vehicle-treated diabetic mice (Fig. 3n, p, r and t).

Notably, AAV-*si-GR* injection deprived FX5 of its above-mentioned therapeutic capability against the corresponding indicators of OFT test (Supplementary Fig. S2c–d), NOR test (Fig. 3g, h), Y-maze test (Fig. 3k, l) and MWM test (Fig. 3q, t) in AAV-*si-GR* injected diabetic mice.

Collectively, all results demonstrated that FX5 ameliorated DCI by antagonizing GR, and such amelioration had no impacts on the levels of blood glucose, body weight and diet in diabetic mice (Supplementary Fig. S2m–x).

### FX5 ameliorated synaptic impairment through GR/BDNF/TrkB/CREB pathway in diabetic mice

Since we have determined the protection of FX5 against synaptic impairment in primary neurons, we next evaluated such a protective effect in mice. Considering that we have determined the regulation of FX5 against GR nuclear translocation in U2OS/GR-GFP cells, we firstly investigated whether FX5 also exhibited such an effect in mice. For this purpose, neurons and microglia in the hippocampus of diabetic mice were identified by MAP2 and Iba1 antibodies [31] respectively, and immunostaining results indicated that FX5 inhibited GR nuclear translocation in brain neurons and microglia of diabetic mice (Supplementary Fig. S3a–h).

#### FX5 protected synaptic integrity by antagonizing GR

**Fig. 4:**
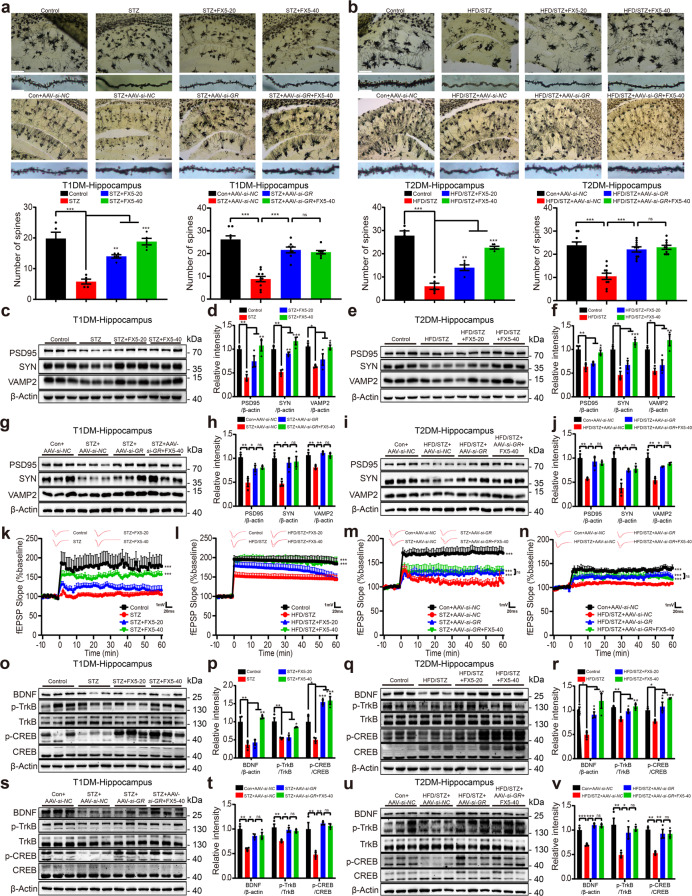
FX5 ameliorated synaptic impairment through GR/BDNF/TrkB/CREB pathway in the hippocampus of diabetic mice. FX5 (20, 40 mg/kg) or AAV-*si-GR* treatment reversed the spine density deficiency of hippocampal neuron in (**a**) STZ and (**b**) HFD/STZ mice, and FX5 (40 mg/kg) had no impacts on such a deficiency in STZ + AAV-*si-GR* and HFD/STZ + AAV-*si-GR* mice (*n* ≥ 5 per group). Scale bar: 200 μm, 10 μm. FX5 (20, 40 mg/kg) or AAV-*si-GR* treatment increased the expression of PSD95, SYN and VAMP2 in the hippocampus of (**c**, **d**, **g**, **h**) STZ and (**e**, **f**, **i**, **j**) HFD/STZ mice, and FX5 (40 mg/kg) rendered no influence on these proteins in (**g**, **h**) STZ + AAV-*si-GR* and (**i**, **j**) HFD/STZ + AAV-*si-GR* mice (*n* = 3 per group). Changes in fEPSP slope were recorded following high frequency stimulation (4 × 100 Hz), and FX5 (20, 40 mg/kg) or AAV-*si-GR* treatment ameliorated LTP impairments in the hippocampal DG region of (**k**, **m**) STZ and (**l**, **n**) HFD/STZ mice, while FX5 (40 mg/kg) had no impacts on LTP impairments in (**m**) STZ + AAV-*si-GR* and (**n**) HFD/STZ + AAV-*si-GR* mice (animal, *n* = 3 per group; brain slice, *n* ≥ 6 per group). FX5 (20, 40 mg/kg) or AAV-*si-GR* treatment upregulated the expressions of BDNF, p-TrkB and p-CREB in the hippocampus of (**o**, **p**, **s**, **t**) STZ and (**q**, **r**, **u**, **v**) HFD/STZ mice, and FX5 (40 mg/kg) had no impacts on these proteins in (**s**, **t**) STZ + AAV-*si-GR* and (**u**, **v**) HFD/STZ + AAV-*si-GR* mice (*n* = 3 per group). β-Actin was used as loading control. *n* = 3 independent experiments. Mean ± SEM. One-way ANOVA followed by Dunnett’s multiple comparison test. *P < 0.05, **P < 0.01, ***P < 0.001 vs model or AAV-*si-NC* treated model group.

Golgi staining assay was applied to evaluate the development of dendrites and the number of dendritic spines of neurons. As shown in Fig. 4a and b, FX5 or AAV-*si-GR* treatment ameliorated synaptic integrity deficiency in the hippocampus of diabetic mice.

In addition, FX5 or AAV-*si-GR* treatment upregulated the levels of synaptic integrity-related proteins PSD95, SYN, and VAMP2 in the hippocampus and cortex of diabetic mice (Fig. 4c–j and Supplementary Fig. S3i–p), and AAV-*si-GR* injection deprived FX5 of its capability in upregulating the levels of these proteins in AAV-*si-GR*-treated diabetic mice (Fig. 4a–j and Supplementary Fig. S3i–p). These results demonstrated that FX5 improved synapse integrity in diabetic mice by antagonizing GR.

#### FX5 ameliorated LTP by antagonizing GR

Given that LTP of synaptic transmission is commonly applied to detect synaptic plasticity [31], LTP in hippocampal DG region was examined. As indicated in Fig. 4k–n, FX5 or AAV-*si-GR* treatment improved LTP induction and maintenance in diabetic mice, and AAV-*si-GR* injection deprived FX5 of its capability in improving synaptic transmission in AAV-*si-GR*-treated diabetic mice. Thus, all results demonstrated that FX5 improved synapse plasticity in diabetic mice by antagonizing GR.

#### FX5 upregulated BDNF/TrkB/CREB pathway by antagonizing GR

Next, we examined the regulation of FX5 treatment against the protein levels of BDNF, p-TrkB and p-CREB in the hippocampus and cortex of diabetic mice like the case in cell-based assays (Fig. 1h, i). As shown in Fig. 4o–v and Supplementary Fig. S3q–x, FX5 or AAV-*si-GR* treatment upregulated the levels of these proteins in diabetic mice, and AAV-*si-GR* deprived FX5 of its capability in upregulating these proteins in AAV-*si-GR*-treated diabetic mice. Thus, all results demonstrated that FX5 treatment upregulated BDNF/TrkB/CREB pathway in diabetic mice by antagonizing GR.

Together, all results demonstrated that FX5 ameliorated synaptic impairment through GR/BDNF/TrkB/CREB pathway in diabetic mice.

### FX5 protected against neuronal apoptosis by repressing GR/PI3K/AKT/GSK3β pathway-mediated tauopathy and subsequent ER stress in diabetic mice

#### FX5 suppressed neuronal apoptosis by antagonizing GR

**Fig. 5:**
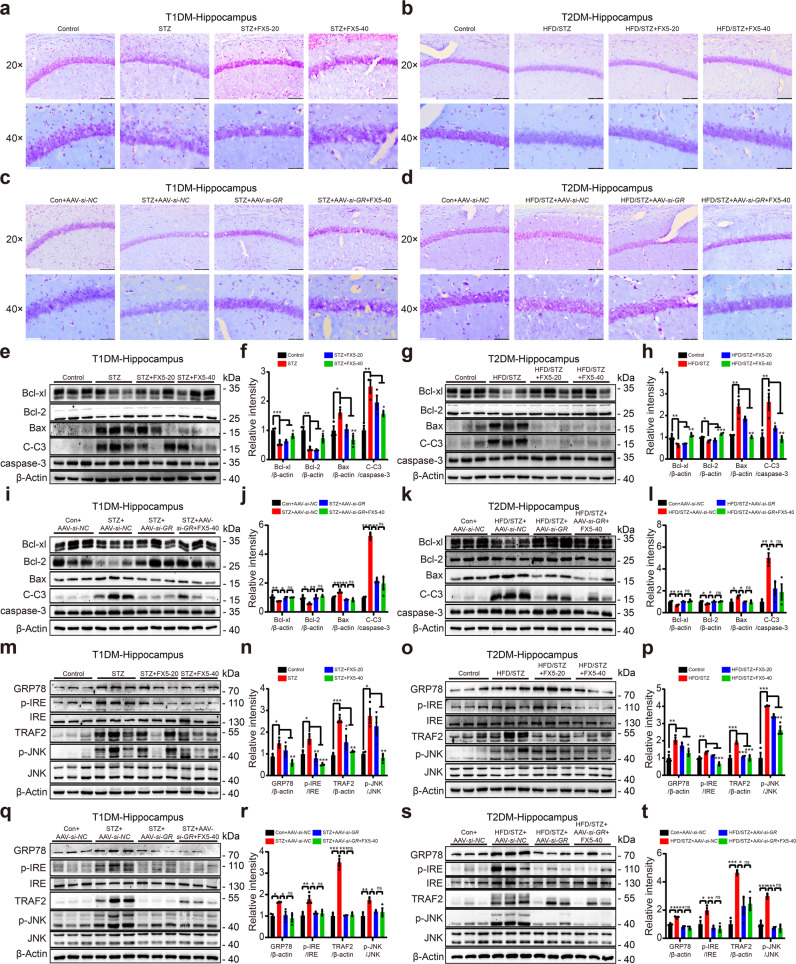
FX5 protected against neuronal apoptosis and ER stress in the hippocampus of diabetic mice. Nissl staining results indicated that FX5 (20, 40 mg/kg) or AAV-*si-GR* treatment increased the number of survival neurons in the hippocampus of (**a**, **c**) STZ and (**b**, **d**) HFD/STZ mice, and FX5 (40 mg/kg) treatment had no such an effect in (**c**) STZ + AAV-*si-GR* and (**d**) HFD/STZ + AAV-*si-GR* mice (*n* = 4 per group). Scale bar: 100 μm, 50 μm. Immunoblot analysis with quantifications showed that FX5 (20, 40 mg/kg) or AAV-*si-GR* treatment suppressed pro-apoptotic process in the hippocampus of (**e**, **f**, **i**, **j**) STZ and (**g**, **h**, **k**, **l**) HFD/STZ mice, and FX5 (40 mg/kg) treatment had no such effects in (**i**, **j**) STZ + AAV-*si-GR* and (**k**, **l**) HFD/STZ + AAV-*si-GR* mice (*n* = 3 per group). Immunoblot analysis with quantifications showed that FX5 (20, 40 mg/kg) or AAV-*si-GR* treatment ameliorated ER stress in the hippocampus of (**m**, **n**, **q**, **r**) STZ and (**o**, **p**, **s**, **t**) HFD/STZ mice, and FX5 (40 mg/kg) treatment had no such an ameliorative effect in (**q**, **r**) STZ + AAV-*si-GR* and (**s**, **t**) HFD/STZ + AAV-*si-GR* mice (*n* = 3 per group). β-Actin was used as loading control. *n* = 3 independent experiments. Mean ± SEM. One-way ANOVA followed by Dunnett’s multiple comparison test. *P < 0.05, **P < 0.01, ***P < 0.001 vs model or AAV-*si-NC* treated model group.

To evaluate the protective effect of FX5 on neurons in mice, we performed Nissl staining and immunoblot assays. The results indicated that the number of survival neurons in CA1 region and the levels of anti-apoptotic proteins Bcl-2 and Bcl-xl were increased, while the levels of pro-apoptotic proteins Bax and C-C3 were decreased in the hippocampus and cortex of FX5 or AAV-*si-GR*-treated diabetic mice compared with those in vehicle-treated diabetic mice, indicative of the capability of FX5 treatment in suppressing neuronal apoptosis. Notably, AAV-*si-GR* treatment deprived FX5 of its above-mentioned capability in AAV-*si-GR*-treated diabetic mice (Fig. 5a–l and Supplementary Fig. S4a–h). These results demonstrated that FX5 suppressed neuronal apoptosis in diabetic mice by antagonizing GR.

#### FX5 repressed ER stress by antagonizing GR

Next, immunoblot assay was performed to investigate whether FX5 treatment repressed ER stress by antagonizing GR in the hippocampus and cortex of diabetic mice. As shown in Fig. 5m–t and Supplementary Fig. S4i–p, FX5 or AAV-*si-GR* treatment suppressed the levels of ER stress-related proteins GRP78, p-IRE, TRAF2 and p-JNK in diabetic mice. Notably, AAV-*si-GR* injection deprived FX5 of its suppressive capability in AAV-*si-GR*-treated diabetic mice. All results demonstrated that FX5 repressed ER stress in diabetic mice by antagonizing GR.

#### FX5 attenuated tauopathy by antagonizing GR

**Fig. 6:**
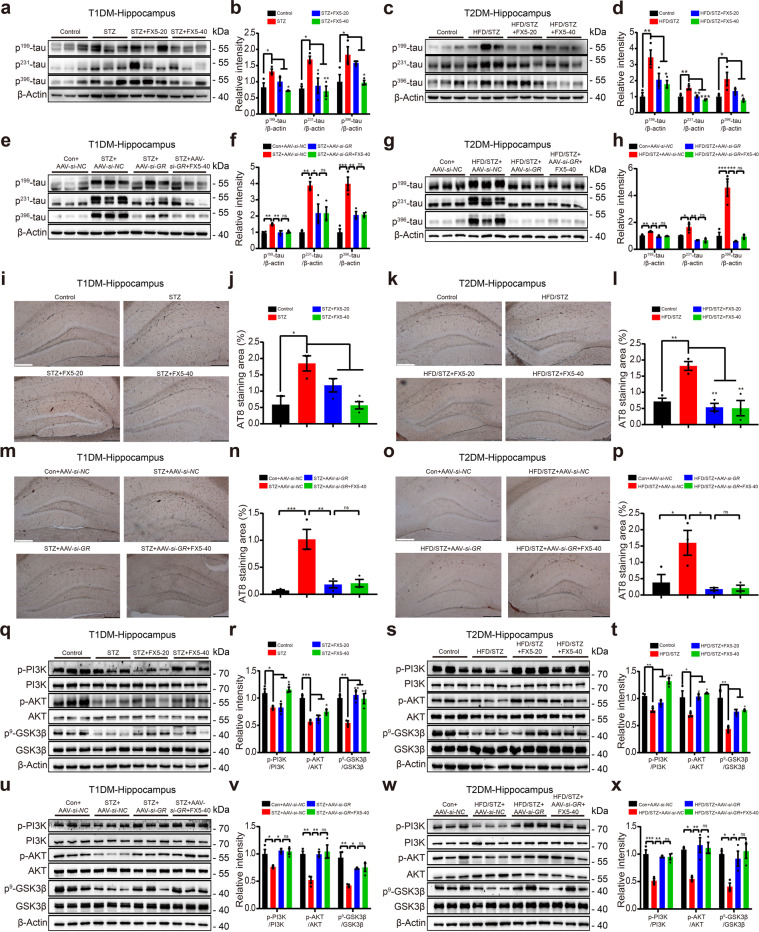
FX5 attenuated tauopathy through GR/PI3K/AKT/GSK3β pathway in the hippocampus of diabetic mice. FX5 (20, 40 mg/kg) or AAV-*si-GR* treatment repressed tau hyperphosphorylation in the hippocampus of (**a**, **b**, **e**, **f**) STZ and (**c**, **d**, **g**, **h**) HFD/STZ mice, and FX5 (40 mg/kg) had no impacts on tau hyperphosphorylation in (**e**, **f**) STZ + AAV-*si-GR* and (**g**, **h**) HFD/STZ + AAV-*si-GR* mice (*n* = 3 per group). FX5 (20, 40 mg/kg) or AAV-*si-GR* treatment suppressed AT8 expression in the hippocampus of (**i**, **j**, **m**, **n**) STZ and (**k**, **l**, **o**, **p**) HFD/STZ mice, and FX5 (40 mg/kg) had no impacts on AT8 expression in (**m**, **n**) STZ + AAV-*si-GR* and (**o**, **p**) HFD/STZ + AAV-*si-GR* mice (positive AT8 protein was labeled brown, *n* = 3 per group). Scale bar: 200 μm. FX5 (20, 40 mg/kg) or AAV-*si-GR* treatment upregulated the levels of p-PI3K, p-AKT (Ser 473) and p-GSK3β (Ser 9) in the hippocampus of (**q**, **r**, **u**, **v**) STZ and (**s**, **t**, **w**, **x**) HFD/STZ mice, and FX5 (40 mg/kg) had no impacts on these proteins in (**u**, **v**) STZ + AAV-*si-GR* and (**w**, **x**) HFD/STZ + AAV-*si-GR* mice (*n* = 3 per group). β-Actin was used as loading control. *n* = 3 independent experiments. Mean ± SEM. One-way ANOVA followed by Dunnett’s multiple comparison test. *P < 0.05, **P < 0.01, ***P < 0.001 vs model or AAV-*si-NC* treated model group.

We next detected the ability of FX5 in attenuating tauopathy by immunoblot assays and DAB staining in the hippocampus and cortex of diabetic mice. As indicated in Fig. 6a–p and Supplementary Fig. S5a–p, FX5 or AAV-*si-GR* treatment decreased the levels of tau phospho-epitopes and AT8 protein, and AAV-*si-GR* treatment deprived FX5 of its above-mentioned capability in AAV-*si-GR*-treated diabetic mice. Therefore, all results demonstrated that FX5 attenuated tauopathy in diabetic mice by antagonizing GR.

#### FX5 regulated PI3K/AKT/GSK3β pathway by antagonizing GR

Like the case in cell-based assay, we examined the levels of p-PI3K, p-AKT (Ser 473) and p-GSK3β (Ser 9) in the hippocampus and cortex of diabetic mice. Immunoblot results suggested that FX5 or AAV-*si-GR* treatment upregulated these phosphorylated proteins in diabetic mice, and AAV-*si-GR* treatment deprived FX5 of its such capability in AAV-*si-GR*-treated diabetic mice (Fig. 6q–x and Supplementary Fig. S5q–x). These results demonstrated that FX5 regulated PI3K/AKT/GSK3β pathway by antagonizing GR in diabetic mice.

Together, FX5 protected against neuronal apoptosis by repressing GR/PI3K/AKT/GSK3β pathway-mediated tauopathy and subsequent ER stress in diabetic mice.

### FX5 repressed inflammation through GR/NF-κB/NLRP3/ASC/Caspase-1 pathway in diabetic mice

#### FX5 treatment ameliorated inflammation by antagonizing GR

**Fig. 7:**
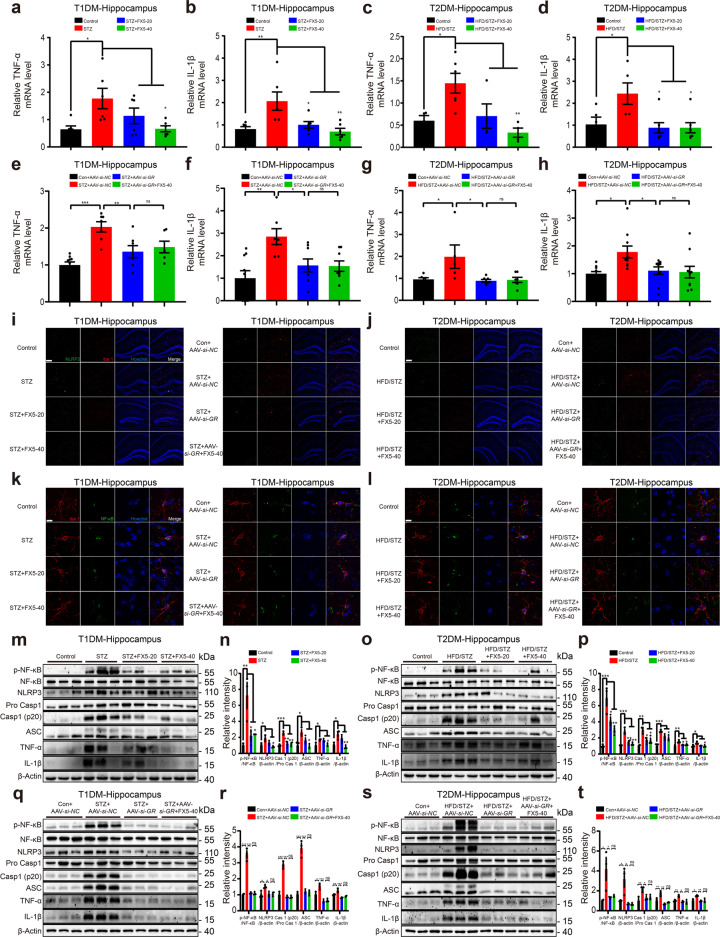
FX5 repressed inflammation through GR/NF-κB/NLRP3/ASC/Caspase-1 pathway in the hippocampus of diabetic mice. FX5 (20, 40 mg/kg) or AAV-*si-GR* treatment attenuated the levels of TNF-α and IL-1β in the hippocampus of (**a**, **b**, **e**, **f**) STZ and (**c**, **d**, **g**, **h**) HFD/STZ mice, and FX5 (40 mg/kg) rendered no such effects in (**e**, **f**) STZ + AAV-*si-GR* and (**g**, **h**) HFD/STZ + AAV-*si-GR* mice (*n* ≥ 4 per group). FX5 (20, 40 mg/kg) or AAV-*si-GR* treatment repressed NLRP3 inflammasome, and FX5 (40 mg/kg) rendered no such an effect in (**i**) STZ + AAV-*si-GR* and (**j**) HFD/STZ + AAV-*si-GR* mice (*n* ≥ 8 per group). Scale bar: 10 μm. FX5 (20, 40 mg/kg) or AAV-*si-GR* treatment suppressed the nuclear translocation of NF-κB in microglia of diabatic mice, and FX5 (40 mg/kg) rendered no such an effect in (**k**) STZ + AAV-*si-GR* and (**l**) HFD/STZ + AAV-*si-GR* mice (*n* ≥ 7 per group). Scale bar: 10 μm. FX5 (20, 40 mg/kg) or AAV-*si-GR* treatment suppressed the protein levels of p-NF-κB, NLRP3, ASC, Caspase-1 (p20), TNF-α and IL-1β in the hippocampus of (**m**, **n**, **q**, **r**) STZ and (**o**, **p**, **s**, **t**) HFD/STZ mice, and FX5 (40 mg/kg) had no such effects in (**q**, **r**) STZ + AAV-*si-GR* and (**s**, **t**) HFD/STZ + AAV-*si-GR* mice (*n* = 3 per group). β-Actin was used as loading control. *n* = 3 independent experiments. Mean ± SEM. One-way ANOVA followed by Dunnett’s multiple comparison test. *P < 0.05, **P < 0.01, ***P < 0.001 vs model or AAV-*si-NC* treated model group.

Like the case in cell-based assay, the levels of inflammatory factors TNF-α and IL-1β in the hippocampus and cortex of diabetic mice were detected by RT-PCR and immunoblot assays. As shown in Fig. 7a–h, m–t and Supplementary Fig. S6a–p, FX5 or AAV-*si-GR* treatment suppressed the levels of these inflammatory factors in diabetic mice, and AAV-*si-GR* treatment deprived FX5 of its suppressive effect in AAV-*si-GR*-injected diabetic mice. These results thus demonstrated that FX5 ameliorated inflammation by antagonizing GR.

#### FX5 repressed microglial NLRP3 inflammasome by antagonizing GR

As FX5 was capable of repressing activation of NLRP3 inflammasome by antagonizing GR in primary microglia (Fig. 2m–p), we next inspected such a capability of FX5 in the hippocampus of diabetic mice. In the assay, microglia were identified by Iba1 antibody. Immunostaining results demonstrated that FX5 or AAV-*si-GR* treatment suppressed NLRP3 positive rate in diabetic mice and AAV-*si-GR* injection deprived FX5 of its suppressive capability in AAV-*si-GR*- injected diabetic mice (Fig. 7i, j and Supplementary Fig. S6q, r).

#### FX5 suppressed microglial NF-κB nuclear translocation by antagonizing GR

Given the tight implication of NF-κB in inflammation, we inspected the potential of FX5 in regulating NF-κB nuclear translocation by immunostaining assay in microglia of the hippocampus of diabetic mice. As shown in Fig. 7k, l and Supplementary Fig. S6s, t, FX5 or AAV-*si-GR* treatment inhibited NF-κB nuclear translocation in diabetic mice, and AAV-*si-GR* deprived FX5 of its inhibitory activity in AAV-*si-GR*-treated diabetic mice.

In addition, immunoblot assay results demonstrated that FX5 or AAV-*si-GR* treatment suppressed the protein levels of p-NF-κB, NLRP3, ASC and Caspase-1 (P20) in the hippocampus and cortex of the diabetic mice, and AAV-*si-GR* injection deprived FX5 of its suppressive capability in AAV-*si-GR*-treated diabetic mice (Fig. 7m–t and Supplementary Fig. S6i–p). These results demonstrated that FX5 repressed NF-κB/NLRP3/ASC/Caspase-1 pathway in diabetic mice by antagonizing GR.

Collectively, FX5 repressed inflammation through GR/NF-κB/NLRP3/ASC/Caspase-1 pathway in diabetic mice.

## Discussion

DCI is a chronic complication of diabetes with complicated pathogenesis. It was reported that GR antagonists can improve DCI in mice by repressing hippocampal Aβ generation, neuroinflammation and apoptotic processes [25, 29, 66], but the detailed mechanisms are much needed. Here, we determined that small molecule FX5 as a non-steroidal GR antagonist effectively ameliorated learning and memory impairment in diabetic mice and the underlying mechanisms have been intensively investigated. Our results have highlighted the potential of FX5 in the treatment of DCI.

In the brain, synaptic plasticity and integrity are critically responsible for cognition. BDNF is a widespread neurotrophin facilitating neuroprotection and synaptic interaction by binding its receptor TrkB, and GR regulates BDNF expression through directly binding to BDNF regulatory sequences [67]. Thus, our finding that FX5 efficiently ameliorated synaptic impairment through GR/BDNF/TrkB/CREB pathway has clearly revealed the underlying mechanism for the beneficial effect of GR antagonist on synaptic protection.

Tau as a major microtubule-associated protein of normal mature neurons aggravates neurotoxicity by excessive phosphorylation resulting in cognitive dysfunction in diabetic mice [68]. According to the published report, GR could decrease PI3K activity in skeletal muscle through a direct inhibitory interaction between PI3K p85 regulatory subunit and GR [69]. In diabetic pathology, GCs suppress the expression and activity of PI3K and AKT [70, 71], triggering the activation of GSK3β that is responsible for tau phosphorylation [72]. Subsequently, tau phosphorylation has been also identified to induce ER stress, as indicated by the study on tau transgenic mice that the interaction between tau and ER membrane impaired ER-associated degradation and activated unfolded protein response (UPR) [73, 74] inducing apoptosis [54]. Here, we determined that FX5 protected against neuronal apoptosis by repressing GR/PI3K/AKT/GSK3β pathway-mediated tauopathy and subsequent ER stress, strongly supporting that antagonizing GR might be a promising therapeutic strategy in treating neuronal apoptosis and its related degenerative diseases.

DCI is highly correlated with inflammation [75]. Although GR agonist exhibits anti-inflammation effect, chronic GCs exposure may increase NF-κB activation [76], inducing the expressions of TNF-α [77] and NLRP3 [78]. Here, FX5 as a non-steroidal GR antagonist was determined to efficiently repress inflammation through GR/NF-κB/NLRP3/ASC/Caspase-1 pathway. To our knowledge, our work might be the first report on the mechanism underlying the regulation of GR against inflammation in DCI.

**Fig. 8:**
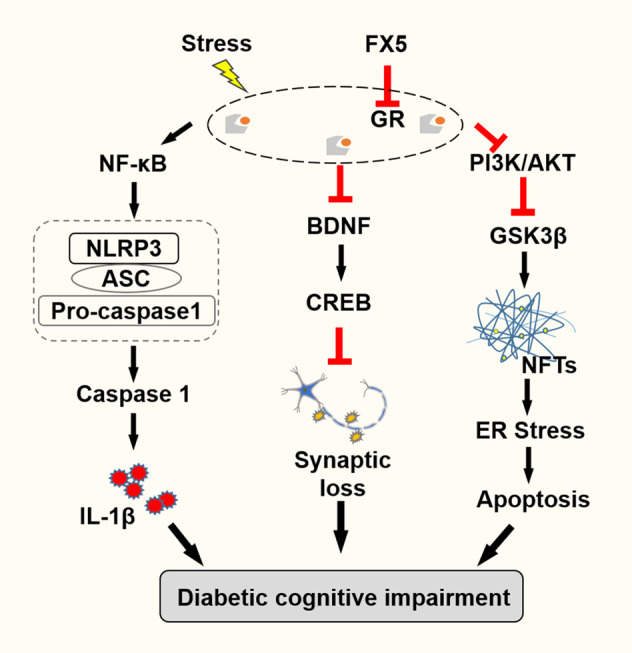
The mechanisms underlying the protective effects of FX5 against diabetic cognitive impairment. FX5 as a non-steroidal GR antagonist ameliorated synaptic impairment through GR/BDNF/TrkB/CREB pathway, protected against neuronal apoptosis by repressing GR/PI3K/AKT/GSK3β pathway-mediated tauopathy and subsequent ER stress, and repressed inflammation through GR/NF-κB/NLRP3/ASC/Caspase-1 pathway.

In summary, we determined that small molecule FX5 as a non-steroidal GR antagonist efficiently ameliorated learning and memory impairment in diabetic mice. The underlying mechanisms have been intensively investigated. As summarized in Fig. 8, FX5 ameliorated synaptic impairment through GR/BDNF/TrkB/CREB pathway, protected against neuronal apoptosis by repressing GR/PI3K/AKT/GSK3β pathway-mediated tauopathy and subsequent ER stress and repressed inflammation through GR/NF-κB/NLRP3/ASC/Caspase-1 pathway. Our work has strongly addressed the potency of non-steroidal GR antagonist in the amelioration of DCI and highlighted the potential of FX5 in the treatment of this disease, although further research (e.g., structure optimization, toxicity, and oral availability evaluation) is much needed before its clinical application.

## Supplementary information

Supplemental Document

Supplementary Figure 1

Supplementary Figure 2

Supplementary Figure 3

Supplementary Figure 4

Supplementary Figure 5

Supplementary Figure 6
