# Supplementary material for: FX5, a non-steroidal glucocorticoid receptor antagonist, ameliorates diabetic cognitive impairment in mice
Source: Acta Pharmacol Sin. 2022 Mar 8;43(10):2495–510. doi: 10.1038/s41401-022-00884-9 (PMC9525278; doi:10.1038/s41401-022-00884-9)
Supplement: Supplementary file 1 — Supplemental Document [file 41401_2022_884_MOESM1_ESM.docx]

**FIGURE LEGENDS**

**Fig. S1 Assays of AAV-*si-GR* efficiency, mammalian transactivation, cell viability, and GR nuclear translocation.** Immunoblot analysis with quantifications showed the efficiency of AAV-*si-GR* in the cortex and hippocampus of **(a, b)** STZ and **(c, d)** HFD/STZ mice (*n* = 3 per group). Mammalian transactivation experiment was carried out to detect the effect of FX5 on **(e)** GR, **(f)** MR, **(g)** PR, **(h)** LXRβ and **(i)** ERβ (*n* = 3 per group). The positive agonists: Dex (10 nM) for GR, Cort (100 nM) for MR, Prog (100 nM) for PR, T090 (5 μM) for LXRβ and Est (100 nM) for ERβ. **(j)** MTT results demonstrated that FX5 had no impacts on cell viability of primary neurons at concentrations of 2, 5, 10 μM (*n* = 3). **(k-m)** Immunostaining with quantification assay results demonstrated that FX5 antagonized PA-induced GR expression and GR nuclear translocation in U2OS/GR-GFP cells (*n* = 8 per group). Scale bar: 25 μm. β-Actin was used as loading control in immunoblot assays. Data were obtained from three independent experiments. Results are mean ± SEM. Unpaired 2-tailed Student's *t*-test was used for two-group comparison. One-way ANOVA followed by Dunnett’s multiple comparison test. **P* < 0.05, ***P* < 0.01, ****P* < 0.001 compared with AAV-*si-NC* treated model group.

**Fig. S2 FX5 ameliorated learning and memory dysfunction, while had no impacts on the levels of bodyweight, food intake and blood glucose of diabetic mice.** OFT results indicated that FX5 (20, 40 mg/kg) or AAV-*si-GR* treatment increased the time spent in center in **(a, c)** STZ and **(b, d)** HFD/STZ mice, and FX5 (40 mg/kg) treatment had no such an effect in **(c)** STZ+AAV-*si-GR* and **(d)** HFD/STZ+AAV-*si-GR* mice (*n* ≥ 8 per group). **(e-h)** FX5 (20, 40 mg/kg) or AAV-*si-GR* treatment had no effects on the totally moved distance in open field (*n* ≥ 10 per group). Total arm entries in Y-maze test indicated that FX5 (20, 40 mg/kg) or AAV-*si-GR* treatment had no impacts on the total arm entries in **(i)** STZ and **(k)** STZ+AAV-*si-GR* mice (*n* ≥ 14 per group). There were no effects on the total arm entries in **(j)** HFD/STZ and **(l)** HFD/STZ+AAV-*si-GR* mice (*n* ≥ 12 per group). Plasma glucose concentration, body weight and food intake of **(m-o)** STZ (*n* ≥ 19), **(p-r)** HFD/STZ (*n* ≥ 15), **(s-u)** STZ+AAV-*si-NC* (*n* ≥ 14) and **(v-x)** HFD/STZ+AAV-*si-NC* (*n* ≥ 16) mice. Results are mean ± SEM. One-way ANOVA followed by Dunnett’s multiple comparison test. **P* < 0.05, ***P* < 0.01, ****P* < 0.001 compared with model or AAV-*si-NC* treated model group.

**Fig. S3 FX5 suppressed GR nuclear translocation in neurons and microglia of diabetic mice and ameliorated synaptic impairment through GR/BDNF/TrkB/CREB pathway in the** **cortex of diabetic mice.** Immunostaining with quantification assay results demonstrated that FX5 (20, 40 mg/kg) treatment suppressed GR nuclear translocation in neurons of **(a, b)** STZ and **(c, d)** HFD/STZ mice (*n* ≥ 8 per group). Scale bar: 10 μm. Immunostaining with quantification assay results demonstrated that FX5 (20, 40 mg/kg) treatment attenuated GR nuclear translocation in microglia of **(e, f)** STZ and **(g, h)** HFD/STZ mice (*n* = 8 per group). Scale bar: 10 μm. Immunoblot analysis with quantifications showed that FX5 (20, 40 mg/kg) or AAV-*si-GR* treatment increased the expression levels of PSD95, SYN and VAMP2 in the cortex of **(i, j, m, n)** STZ and **(k, l, o, p)** HFD/STZ mice, and FX5 (40 mg/kg) treatment had no such effects in **(m, n)** STZ+AAV-*si-GR* and **(o, p)** HFD/STZ+AAV-*si-GR* mice (*n* = 3 per group). Immunoblot analysis with quantifications showed that FX5 (20, 40 mg/kg) or AAV-*si-GR* treatment elevated the expression levels of BDNF, p-TrkB and p-CREB in the cortex of **(q, r, u, v)** STZ and **(s, t, w, x)** HFD/STZ mice, and FX5 (40 mg/kg) treatment had no such effects in **(u, v)** STZ+AAV-*si-GR* and **(w, x)** HFD/STZ+AAV-*si-GR* mice (*n* = 3 per group). β-Actin was used as loading control in immunoblot assays. Data were obtained from three independent experiments. Results are mean ± SEM. One-way ANOVA followed by Dunnett’s multiple comparison test. **P* < 0.05, ***P* < 0.01, ****P* < 0.001 compared with model or AAV-*si-NC* treated model group.

**Fig. S4 FX5 protected against neuronal apoptosis and ER stress in the cortex of diabetic mice.** Immunoblot analysis with quantifications showed that FX5 (20, 40 mg/kg) or AAV-*si-GR* treatment suppressed pro-apoptotic process in the cortex of **(a, b, e, f)** STZ and **(c, d, g, h)** HFD/STZ mice, and FX5 (40 mg/kg) treatment had no such an effect in **(e, f)** STZ+AAV-*si-GR* and **(g, h)** HFD/STZ+AAV-*si-GR* mice (*n* = 3 per group). Immunoblot analysis with quantifications showed that FX5 (20, 40 mg/kg) or AAV-*si-GR* treatment ameliorated ER stress in the cortex of **(i, j, m, n)** STZ and **(k, l, o, p)** HFD/STZ mice, and FX5 (40 mg/kg) treatment had no such an effect in **(m, n)** STZ+AAV-*si-GR* and **(o, p)** HFD/STZ+AAV-*si-GR* mice (*n* = 3 per group). β-Actin was used as loading control in immunoblot assays. Data were obtained from three independent experiments. Results are mean ± SEM. One-way ANOVA followed by Dunnett’s multiple comparison test. **P* < 0.05, ***P* < 0.01, ****P* < 0.001 compared with model or AAV-*si-NC* treated model group.

**Fig. S5 FX5 attenuated tauopathy through GR/PI3K/AKT/GSK3β pathway in the cortex of diabetic mice.** Immunoblot analysis with quantifications showed that FX5 (20, 40 mg/kg) or AAV-*si-GR* treatment suppressed tau hyperphosphorylation in the cortex of **(a, b, e, f)** STZ and **(c, d, g, h)** HFD/STZ mice, and FX5 (40 mg/kg) treatment had no such an effect in **(e, f)** STZ+AAV-*si-GR* and **(g, h)** HFD/STZ+AAV-*si-GR* mice (*n* = 3 per group). DAB staining and its quantification results indicated that FX5 (20, 40 mg/kg) or AAV-*si-GR* treatment suppressed AT8 expression in the cortex of **(i, j, m, n)** STZ and **(k, l, o, p)** HFD/STZ mice, while FX5 (40 mg/kg) treatment had no such an effect in **(m, n)** STZ+AAV-*si-GR* and **(o, p)** HFD/STZ+AAV-*si-GR* mice (*n* ≥ 6 per group). Scale bar: 200 μm. Immunoblot analysis with quantifications showed that FX5 (20, 40 mg/kg) or AAV-*si-GR* treatment upregulated the levels of p-PI3K, p-AKT (Ser 473) and p-GSK3β (Ser 9) in the cortex of **(q, r, u, v)** STZ and **(s, t, w, x)** HFD/STZ mice, and FX5 (40 mg/kg) treatment had no such an effect in **(u, v)** STZ+AAV-*si-GR* and **(w, x)** HFD/STZ+AAV-*si-GR* mice (*n* = 3 per group). β-Actin was used as loading control in immunoblot assays. Data were obtained from three independent experiments. Results are mean ± SEM. One-way ANOVA followed by Dunnett’s multiple comparison test. **P* < 0.05, ***P* < 0.01, ****P* < 0.001 compared with model or AAV-*si-NC* treated model group.

**Fig. S6 FX5 repressed inflammation through GR/NF-κB/NLRP3/ASC/Caspase-1 pathway in the cortex of diabetic mice.** RT-PCR results demonstrated that FX5 (20, 40 mg/kg) or AAV-*si-GR* treatment attenuated the levels of inflammatory factors TNF-α and IL-1β in the cortex of **(a, b, e, f)** STZ and **(c, d, g, h)** HFD/STZ mice, and FX5 (40 mg/kg) treatment had no such an effect in **(e, f)** STZ+AAV-*si-GR* and **(g, h)** HFD/STZ+AAV-*si-GR* mice (*n* ≥ 5 per group). Immunoblot analysis with quantifications showed that FX5 (20, 40 mg/kg) or AAV-*si-GR* treatment suppressed the protein levels of p-NF-κB, NLRP3, ASC, Caspase-1 (p20), TNF-α and IL-1β in the cortex of **(i, j, m, n)** STZ and **(k, l, o, p)** HFD/STZ mice, and FX5 (40 mg/kg) treatment had no such an effect in **(m, n)** STZ+AAV-*si-GR* and **(o, p)** HFD/STZ+AAV-*si-GR* mice (*n* = 3 per group). Quantification results of NLRP3 expression in **(q)** STZ or STZ+AAV-*si-GR* and **(r)** HFD/STZ or HFD/STZ+AAV-*si-GR* mice. Quantification results of NF-κB translocation assay in **(s)** STZ or STZ+AAV-*si-GR* and **(t)** HFD/STZ or HFD/STZ+AAV-*si-GR* mice. β-Actin was used as loading control in immunoblot assays. Data were obtained from three independent experiments. Results are mean ± SEM. One-way ANOVA followed by Dunnett’s multiple comparison test. **P* < 0.05, ***P* < 0.01, ****P* < 0.001 compared with model or AAV-*si-NC* treated model group.

**SUPPLEMENTAL DATA**

**Supplementary Table 1. Antibodies used for Immunoblot assay.**

| **Resource** | **Source** | **Identifier** | **RRID** |
| --- | --- | --- | --- |
| Rabbit anti**-**Synaptophysin | Cell Signaling Technology | Cat# 5461 | AB_10698743 |
| Rabbit anti-PSD95 | Cell Signaling Technology | Cat# 3409 | AB_1264242 |
| Rabbit anti-VAMP2 | Cell Signaling Technology | Cat# 13508 | AB_2798240 |
| Rabbit anti-BDNF | Abcam | Cat# ab108319 | AB_10862052 |
| Rabbit anti-p-TrkB | Abcam | Cat# ab229908 | AB_2892153 |
| Rabbit anti-TrkB | Cell Signaling Technology | Cat# 4603 | AB_2155125 |
| Rabbit anti-p-CREB | Cell Signaling Technology | Cat# 9198 | AB_2561044 |
| Rabbit anti-CREB | Cell Signaling Technology | Cat# 9197 | AB_331277 |
| Rabbit anti-tau (phospho S199) | Abcam | Cat# ab81268 | AB_1641106 |
| Rabbit anti-tau (phospho T231) | Abcam | Cat# ab151559 | AB_2893278 |
| Rabbit anti-tau (phospho S396) | Abcam | Cat# ab109390 | AB_10860822 |
| Rabbit anti-p-PI3K | Cell Signaling Technology | Cat# 4228 | AB_659940 |
| Rabbit anti-PI3K | Cell Signaling Technology | Cat# 4257 | AB_659889 |
| Rabbit anti-p-AKT | Cell Signaling Technology | Cat# 13038 | AB_2629447 |
| Rabbit anti-AKT | Cell Signaling Technology | Cat# 9272 | AB_329827 |
| Rabbit anti-p-GSK3β (Ser9) | Cell Signaling Technology | Cat# 9322 | AB_2115196 |
| Rabbit anti-GSK3β | Cell Signaling Technology | Cat# 12456 | AB_2636978 |
| Rabbit anti-Bcl-xl | Cell Signaling Technology | Cat# 2764 | AB_2228008 |
| Rabbit anti-Bcl-2 | Cell Signaling Technology | Cat# 3498 | AB_1903907 |
| Rabbit anti-Bcl-2 | Abcam | Cat# ab32124 | AB_725644 |
| Rabbit anti-Bax | Cell Signaling Technology | Cat# 2772 | AB_10695870 |
| Rabbit anti-Cleaved caspase-3 | Cell Signaling Technology | Cat# 9661 | AB_2341188 |
| Rabbit anti-Caspase-3 | Cell Signaling Technology | Cat# 9665 | AB_2069872 |
| Mouse anti-GRP78 | BD Biosciences | Cat# 610978 | AB_398291 |
| Rabbit anti-p-IRE | Abcam, | Cat# ab48187 | AB_873899 |
| Rabbit anti-IRE | Cell Signaling Technology | Cat# 3294 | AB_823545 |
| Rabbit anti-TRAF2 | Cell Signaling Technology | Cat# 4712 | AB_2209848 |
| Rabbit anti-p-JNK | Cell Signaling Technology | Cat# 4671 | AB_331338 |
| Rabbit anti-JNK | Cell Signaling Technology | Cat# 9252 | AB_2250373 |
| Rabbit anti-IL-1β | Cell Signaling Technology | Cat# 31202 | AB_2799001 |
| Rabbit anti-TNF-α | Cell Signaling Technology | Cat# 11948 | AB_2687962 |
| Rabbit anti-p-NF-κB | Cell Signaling Technology | Cat# 3033 | AB_331284 |
| Rabbit anti-NF-κB | Cell Signaling Technology | Cat# 8242 | AB_10859369 |
| Rabbit anti-NLRP3 | Cell Signaling Technology | Cat# 15101 | AB_2722591 |
| Rabbit anti-ASC | Cell Signaling Technology | Cat# 67824 | AB_2799736 |
| Mouse anti-Caspase-1 (p20) | AdipoGen | Cat# AG-20B-0042 | AB_2490248 |
| Mouse anti- GAPDH | Proteintech | Cat# 60004-1-lg | AB_2107436 |
| Mouse anti-β-Actin | Abcam | Cat# ab8226 | AB_306371 |
| Rabbit anti-Glucocorticoid receptor | Cell Signaling Technology | Cat# 12041 | AB_2631286 |

**Supplementary Table 2. List of mouse primer sequences (5’-3’) for RT-PCR assay.**

| **Primer** | **Forward** | **Reverse** |
| --- | --- | --- |
| TNF-α | CCCACGTCGTAGCAAACCACCA | CCATTGGCCAGGAGGGCGTTG |
| IL-1β | GCAGCAGCACATCAACAAGAGC | TGTCCTCATCCTGGAAGGTCCACG |
| NLRP3 | GTGGTGACCCTCTGTGAGGT | TCTTCCTGGAGCGCTTCTAA |
| GAPDH | ACAGCAACAGGGTGGTGGAC | TTTGAGGGTGCAGCGAACTT |
| β-Actin | GGCTGTATTCCCCTCCATCG | CCAGTTGGTAACAATGCCATGT |

**Supplementary Table 3. Antibodies used for Immunostaining.**

| **Resource** | **Source** | **Identifier** | **RRID** |
| --- | --- | --- | --- |
| Rabbit anti-VAMP2 | Cell Signaling Technology | Cat# 13508 | AB_2798240 |
| Rabbit anti-PSD95 | Cell Signaling Technology | Cat# 3409 | AB_1264242 |
| Rabbit anti-Glucocorticoid receptor | Cell Signaling Technology | Cat# 12041 | AB_2631286 |
| Rabbit anti-MAP2 | Cell Signaling Technology | Cat# 4542 | AB_10693782 |
| Rabbit anti-Iba 1 | FUJIFILM Wako Shibayagi | Cat# 019-19741 | AB_839504 |
| Rabbit anti-NF-κB | Cell Signaling Technology | Cat# 8242 | AB_10859369 |
| Goat anti-NLRP3 | Abcam | Cat# ab4207 | AB_955792 |
