# Supplementary figures and images for: FX5, a non-steroidal glucocorticoid receptor antagonist, ameliorates diabetic cognitive impairment in mice
Source: Acta Pharmacol Sin. 2022 Mar 8;43(10):2495–510. doi: 10.1038/s41401-022-00884-9 (PMC9525278; doi:10.1038/s41401-022-00884-9)

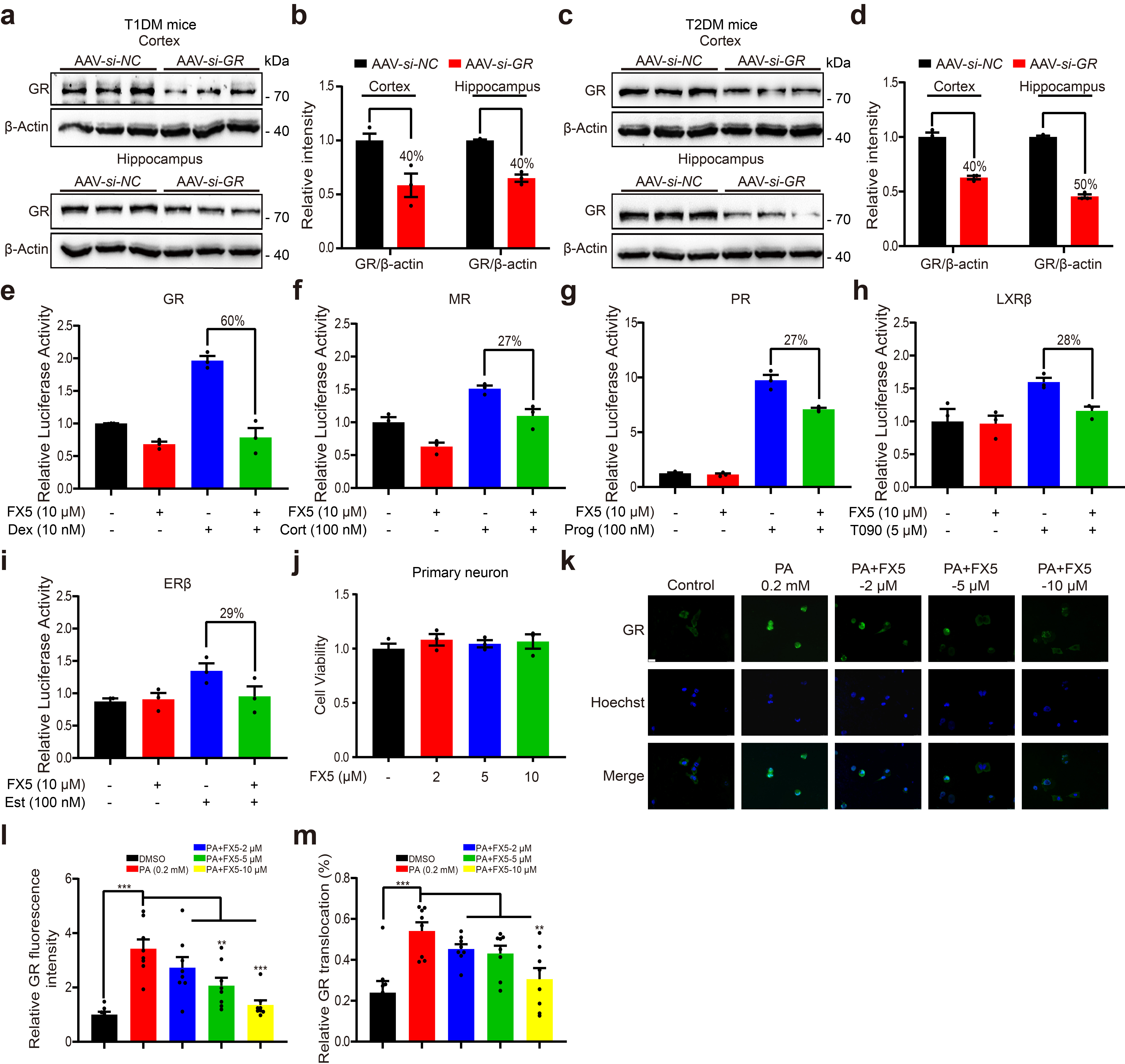

Supplement: Supplementary file 2 — Supplementary Figure 1 [file 41401_2022_884_MOESM2_ESM.jpg]

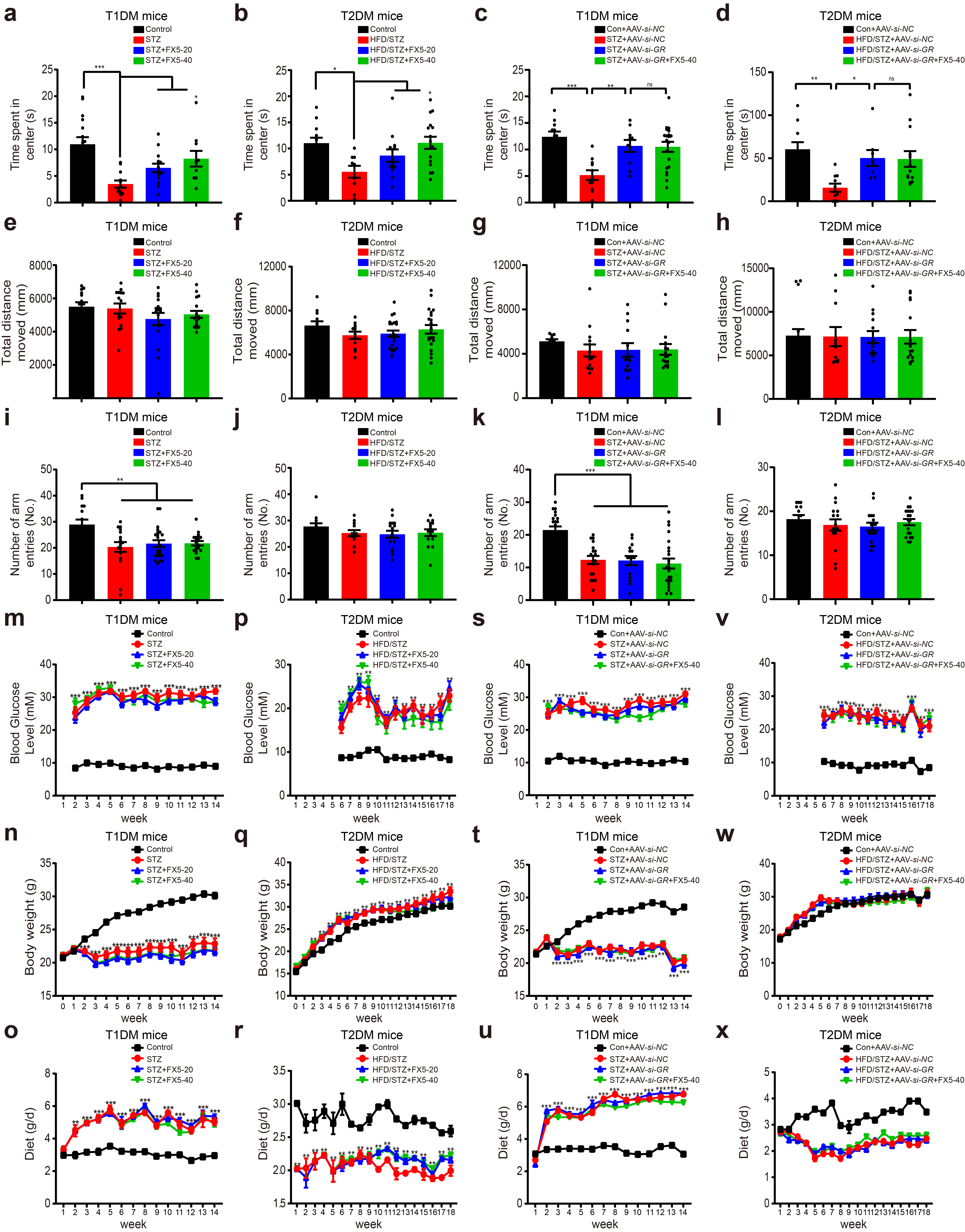

Supplement: Supplementary file 3 — Supplementary Figure 2 [file 41401_2022_884_MOESM3_ESM.jpg]

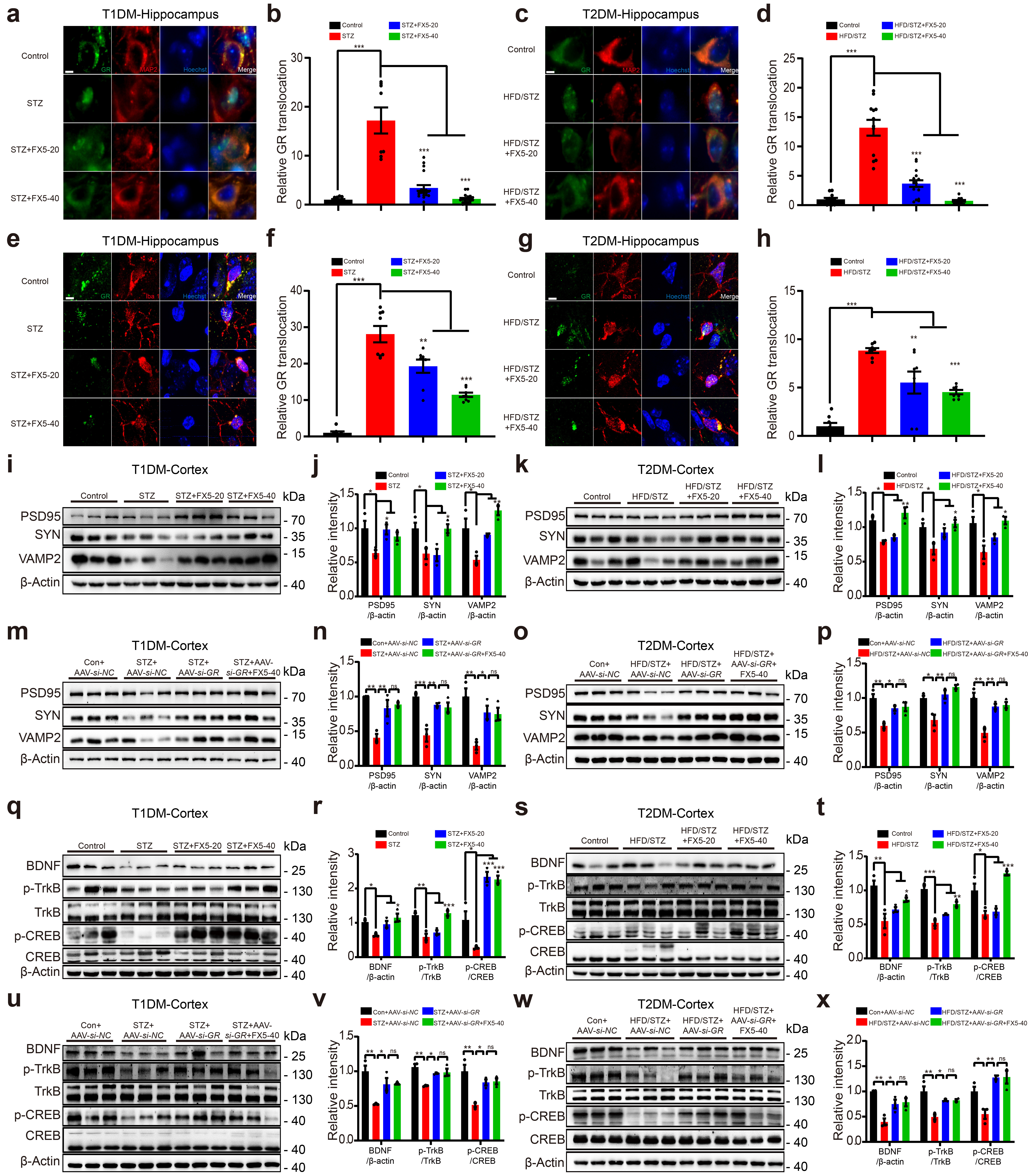

Supplement: Supplementary file 4 — Supplementary Figure 3 [file 41401_2022_884_MOESM4_ESM.jpg]

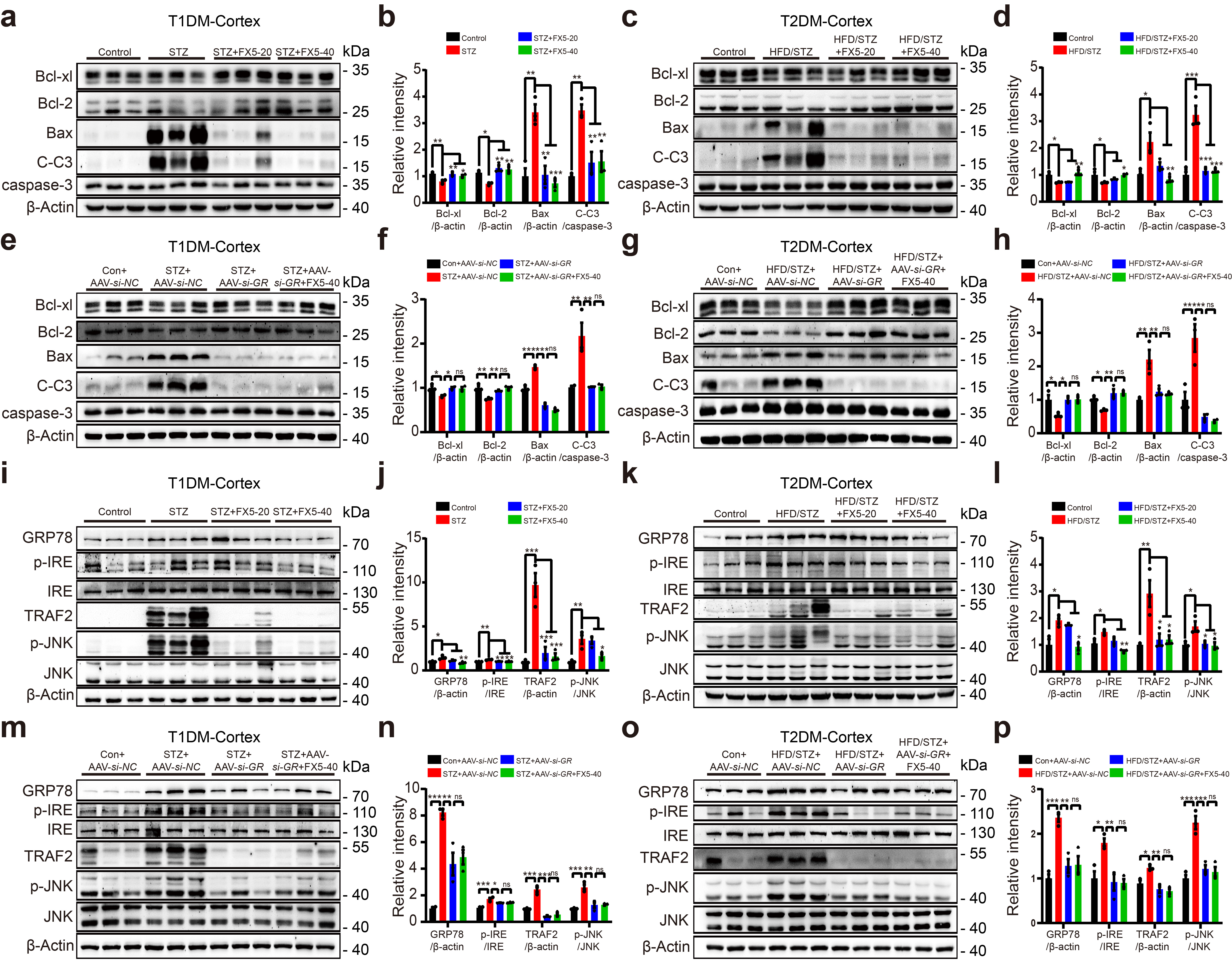

Supplement: Supplementary file 5 — Supplementary Figure 4 [file 41401_2022_884_MOESM5_ESM.jpg]

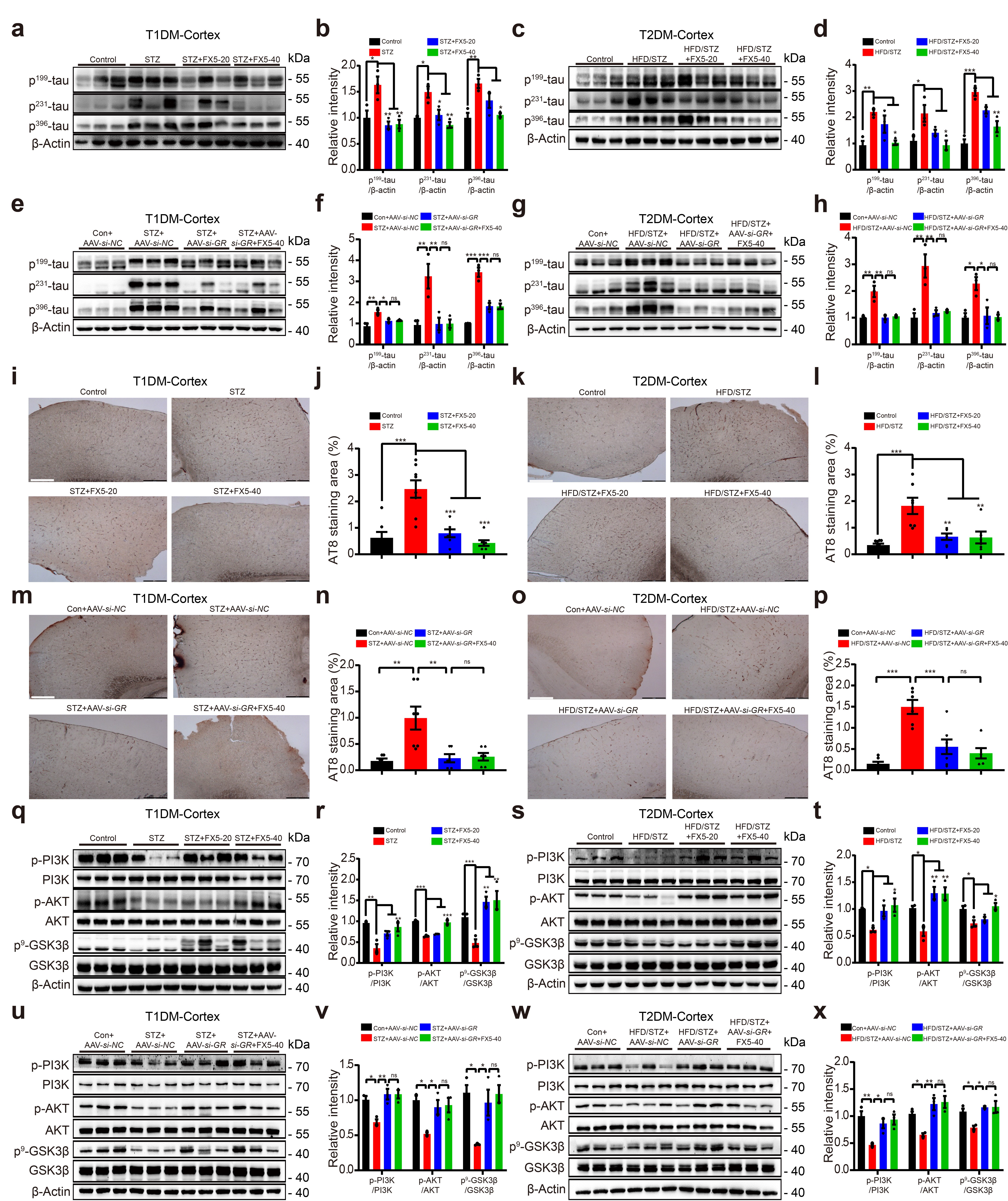

Supplement: Supplementary file 6 — Supplementary Figure 5 [file 41401_2022_884_MOESM6_ESM.jpg]

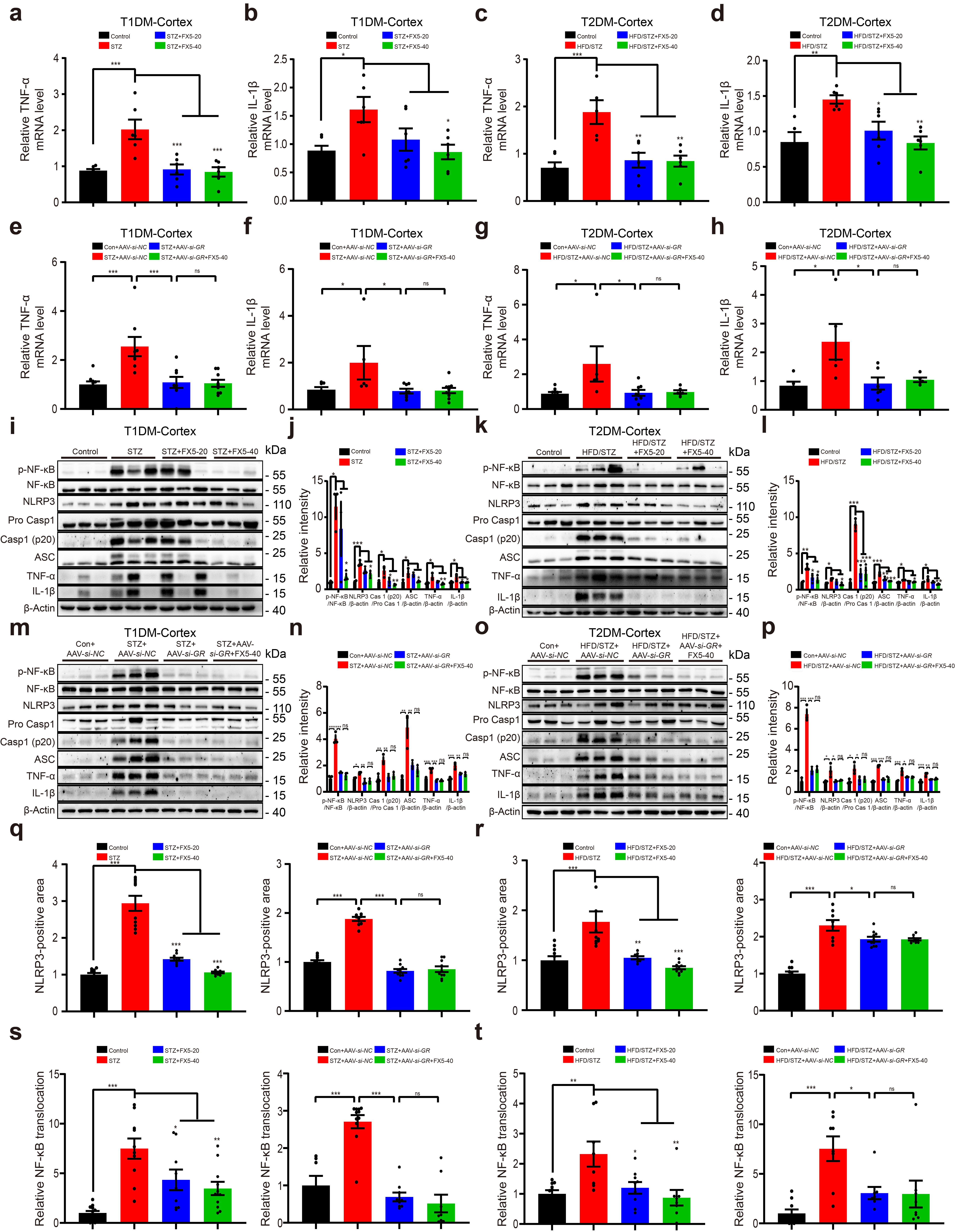

Supplement: Supplementary file 7 — Supplementary Figure 6 [file 41401_2022_884_MOESM7_ESM.jpg]
